# *Hylocereus undatus* flower inhibits lipopolysaccharide-induced acute lung injury in mice by regulating the gut-lung axis and inflammatory response

**DOI:** 10.3389/fphar.2026.1803336

**Published:** 2026-06-15

**Authors:** Shujie Fu, Wanzhong Liao, Bizuan He, Huafeng Wu, Jing Huang, Weizhe Jiang

**Affiliations:** 1 Pharmaceutical College, Guangxi Medical University, Nanning, China; 2 Guangxi Key Laboratory of Bioactive Molecules Research and Evaluation, Nanning, China; 3 College of Biological and Chemical Engineering, Guangxi University of Science and Technology, Liuzhou, China; 4 Department of Pharmacy, Affiliated Hospital of Youjiang Medical University for Nationalities, Baise, China; 5 Key Laboratory of Metabolic Diseases of Baise, Baise, China

**Keywords:** acute lung injury, gut microbiota, gut–lung axis, hylocereus undatus flower, short-chain fatty acids

## Abstract

**Background:**

*Hylocereus undatus* flower (HUF), the dried flower of *H. undatus* (Haw.) Britton and Rose, was first recorded in the Lingnan Record of Medicinal Herbs and is now listed in the Dictionary of Traditional Chinese Medicines. As an established traditional remedy, HUF is primarily used to treat various pulmonary conditions, including pneumonia, asthma, and tuberculosis. Acute lung injury (ALI) represents a critical and common pathological manifestation underlying severe respiratory disorders. However, the potential protective effects of HUF against ALI, and the specific mechanisms involved, remain unelucidated.

**Purpose:**

To systematically evaluate the protective effects of HUF extract on ALI and the pharmacological mechanisms involved.

**Methods:**

Using a lipopolysaccharide-induced mouse model of ALI, we investigated the effects of HUF on the intestinal microbiota of ALI mice via microbial sequencing, metabolomics sequencing, and fecal microbiota transplantation (FMT). We explored the targets and signaling pathways of HUF intervention using transcriptomics and network pharmacology methods and then validated our results using immunological experiments and polymerase chain reaction analysis (PCR).

**Results:**

HUF reduced lung indices and wet-dry ratios associated with pulmonary edema, alleviated inflammatory responses, and mitigated the severity of pulmonary pathological damage. HUF also increased the expression of intestinal tight junction protein (ZO-1), modulated the structure and relative abundance of the gut microbiota, and elevated the levels of certain short-chain fatty acids (SCFAs). FMT experiments confirmed that the HUF-mediated remodeling of the microbiota played a significant role in alleviating ALI. Transcriptomics and network pharmacology analyses suggested that the mechanism underling the interventional effect of HUF on ALI is related to inflammatory pathways. Immunological and PCR experiments further confirmed an association with the P38 mitogen-activated protein kinase (MAPK) pathway.

**Conclusion:**

HUF is associated with attenuation of ALI, correlating with modulation of the gut-lung axis and the inflammatory pathways, and HUF may be a candidate for the treatment of ALI.

## 1 Introduction

Acute lung injury (ALI) is a severe clinical syndrome characterized by hypoxemia, diffuse pulmonary edema, and an overwhelming inflammatory response in the alveolar–capillary membrane (Hughes and Beasley, 2017). Despite advances in supportive care and mechanical ventilation strategies, the mortality rate associated with ALI and its more severe form, remains high, highlighting the urgent requirement for novel therapeutic interventions.

Recent evidence has suggested that the lung and gut are physiologically and pathologically interconnected, forming a close bidirectional regulatory network via immune, metabolic, and neural pathways (Chioma et al., 2022). Disruption of the gut barrier and disturbances in the gut flora can induce the development of lung disease via the gut–lung axis (Budden et al., 2017). The gut, as the largest immune organ and the primary reservoir of the microbiota, plays a crucial role in maintaining pulmonary homeostasis. Under physiological conditions, the gut microbiota indirectly suppresses excessive pulmonary inflammation by promoting immune cell maturation and synthesizing anti-inflammatory metabolites (Dickson et al., 2016; Verma et al., 2024). Conversely, during lung infection or injury, inflammatory signals can adversely impact the composition of the gut microbiota through the systemic circulation, thus establishing a vicious cycle of lung injury–gut dysbiosis (Dickson et al., 2016; Dong et al., 2024; Ziaka and Exadaktylos, 2024). Specifically, dysbiosis of the gut microbiota is a central driver of gut–lung axis dysfunction and a key contributor to the progression of ALI. Previous research utilized alipopolysaccharide (LPS)-induced model of ALI and showed that the diversity of the gut microbiota was significantly reduced and the community structure was imbalanced (Tang et al., 2021). This dysbiosis is known to compromise the integrity of the intestinal barrier, increasing permeability and allowing proinflammatory molecules, such as LPS, to translocate into the systemic circulation via the compromised gut–lung axis, ultimately exacerbating lung damage (Zhou and Liao, 2021). Furthermore, the products secreted by the gut microbiota, such as short-chain fatty acids (SCFAs), serve as key signaling molecules in the gut–lung axis, and their regulatory role in pulmonary inflammation has been extensively studied and validated. For instance, SCFAs reach the lungs via the systemic circulation, where they modulate the chemotaxis of neutrophils, suppress the activation of inflammatory cascades, such as the p38 mitogen-activated protein kinase (MAPK) and nuclear factor-kappa B pathways, and reduce the release of proinflammatory cytokines, including tumor necrosis factor-alpha (TNF-α) and interleukin (IL)-6 (Kabel et al., 2016; Yu et al., 2025). Collectively, these findings suggest that regulating the intestinal microbiota and its metabolites may represent an important target for improving ALI.

*Hylocereus undatus* flower (HUF), the dried flower of *H. undatus* (Haw.) Britton and Rose, is rich in minerals, dietary fiber, and amino acids, thus providing considerable nutritional and medicinal value. In China, HUF is traditionally consumed in soups to clear heat, moisten the lungs, and relieve cough and phlegm. Thus far, HUF has been shown to alleviate several pathological lung conditions, including allergic asthma, cough, and tuberculosis (Fu et al., 2014; Liao et al., 2022a). Furthermore, extracts and specific fractions of HUF have been shown to exert protective activities against ALI-related symptoms. For example, an ethanol extract prepared from HUF was shown to suppress inflammatory responses, whereas its polysaccharide fraction exhibited notable antioxidant properties (Liao et al., 2022b; Li et al., 2023). Notably, HUF is rich in soluble dietary fiber, an important substrate for the production of SCFAs in the gut. SCFAs have been reported to attenuate lung injury in models of inflammation (Hildebrand et al., 2023). In our previous work, we revealed that a water extract of HUF significantly reduced inflammatory cell infiltration in the airway and serum levels of proinflammatory factor in models of asthma via a mechanism closely related to the inhibition of inflammatory pathway activation (Liao et al., 2022a). We also demonstrated that HUF can ameliorate imbalances in the gut and enhance intestinal barrier function, thus indicating its strong potential to exert therapeutic effects via the gut–lung axis (Liao et al., 2023).

Although preliminary evidence suggests that HUF exerts a protective effect against lung injury, the specific targets and mechanisms underlying its therapeutic effects on ALI have yet to be elucidated, particularly in relation to regulatory effects on the lung–gut axis. Therefore, in the present study, we systematically analyzed the effects of HUF on ALI and the underlying pharmacological mechanisms by integrating 16S rRNA gut microbiota sequencing, metabolomics analysis, network pharmacology prediction, transcriptomics analysis, and biological validation.

## 2 Materials and methods

### 2.1 Preparation of HUF extract

HUF was crushed and extracted three times with 12 volumes of water (1 h per extraction). Extracts were combined, concentrated under reduced pressure, dried, and crushed to obtain a powdered HUF extract (yield: 34.30%). The herb was formally identified as being authentic by Professor Yangjiao Xie and a sample was retained in the School of Pharmacy at Guangxi Medical University (Reference: D2023062201). For *in vivo* animal experiments, the powdered HUF extract was dissolved in saline at the required dose for administration. The same batch of HUF extract (Reference: 2023050601) was used for all animal experiments. HUF was harvested in May 2023 from the Yulin City area of southern China.

### 2.2 Quality analysis of HUF by high-performance liquid chromatography (HPLC)

Quality control analysis followed the herbal standards established by our research group previously (Liao et al., 2022a; Liao et al., 2022c). In brief, 0.75 g of HUF powder was refluxed with 40 mL of methanol and 10 mL of dilute hydrochloric acid at 80 °C for 30 min. After cooling to room temperature, the mixture was then filtered to yield a sample solution for HPLC analysis. We used a Shimadzu LC-20 A T HPLC system equipped with an Xtimate C18 column (4.6 mm × 250 mm; 5 μm); the mobile phase consisted of 53% methanol and 47% 0.4% phosphoric acid. The elution program consisted of isocratic elution for 30 min. Separation was performed at a column temperature of 30 °C, with an injection volume of 10 μL, a detection wavelength of 362 nm, and a flow rate of 1 mL/min. We used kaempferol and isorhamnetin as quality control substances and the relative contents of these compounds in test samples were determined using the external standard method.

### 2.3 HUF extraction analysis

HUF samples were analyzed by LC-MS (Liquid chromatography–mass spectrometry) in accordance with a previously published protocol (Liao et al., 2023). For this work, we used a LC-MS system consisting of an ACQUITY UPLC I-Class HF ultra-high-performance liquid chromatograph coupled with a tandem QE high-resolution mass spectrometer. We utilized an ACQUITY UPLC HSS T3 column (100 mm × 2.1 mm, 1.8 μm); mobile phase: A-water (containing 0.1% formic acid), B-acetonitrile; see Supplementary Table 1 for the detailed elution program. Flow rate was 0.35 mL/min. Specific mass spectrometry conditions are provided in Supplementary Table 2. Total ion current (TIC) spectra were acquired in positive and negative ion modes and data were processed with Progenesis QI v3.0 software. Compounds with a mass deviation <5 ppm were preliminarily screened and identified by cross-referencing with databases such as PubChem, MassBank, and LuMet-TCM, as well as relevant literature. Compound identification was based on exact mass, secondary fragments, and isotopic distribution, with analysis performed using the LuMet-TCM database developed by Ouyi Biotechnology Co., Ltd. This database was established by uniformly uploading data from the analysis of authentic standards. No absolute quantification was performed for any of the compounds.

### 2.4 Animals

Male C57BL/6 mice aged 6–8 weeks were selected and maintained at the Animal Care Centre of Guangxi Medical University School of Pharmacy in a controlled room environment of 25 °C ± 2 °C, 55%–65% relative humidity, 12 h light and 12 h dark; standard food and sterile water were available *ad libitum.* The experimental animal protocol was performed in strict accordance with requirements approved by the Animal Ethics Committee of Guangxi Medical University (with internationally accepted principles; ethical approval no: SYXK-GUI- 2020–0,004).

At the conclusion of the experiment, mice were euthanized to collect tissue samples. Specifically, mice were deeply anesthetized by an intraperitoneal injection of chloral hydrate (400 mg/kg). After confirming the absence of basic reflexes, euthanasia was performed by cervical dislocation. This protocol strictly adhered to the Guangxi Medical University Animal Euthanasia Guidelines and was approved by the university’s Animal Care and Use Committee.

### 2.5 Animal models

Thirty-five mice were divided into five groups: a normal control (the NC group); a disease control (the LPS group); a positive control group administered with 5 mg/kg of dexamethasone (DEX); a 3 g/kg HUF group (HUF-L); and a 6 g/kg HUF (HUF-H) group; seven mice were allocated to each group. On days 1–7 of the experiment, each group received their corresponding treatment by oral gavage. The dose of DEX was selected based on previously published studies demonstrating its efficacy in LPS-induced models of ALI in mice (Rao et al., 2022). The HUF doses were determined by body surface area-based conversion from the clinically recommended human daily dose and further optimized based on our preliminary dose-ranging experiments (Liao et al., 2023). The NC and LPS groups were given saline as a control at a dose of 0.2 mL/10 g once daily. On day 7 of the experiment, 6 h after administration, each group of mice was injected with 5 mg/kg of LPS (*E. coli* 055:B5, Sigma Aldrich) into the trachea to induce ALI (in the NC group, LPS was replaced by saline solution) (Yang et al., 2020; Wang et al., 2022). Twenty-four hours later, the mice were anesthetized with chloral hydrate and euthanized for analysis.

### 2.6 Fecal microbiota transplantation

Fecal microbiota transplantation (FMT) experiments were performed in accordance with a previous protocol, with slight modifications (Zhu et al., 2022; Yan et al., 2023). C57BL/6 mice were divided into four groups (NC group, the LPS group, the FMT + LPS group, and FMT + LPS + HUF-H group). The treatment protocols for each group are shown in Supplementary Figure 1. With the exception of the NC group, all other groups were administered a mixture of antibiotics for 14 days (0.5 g/L of vancomycin, 1 g/L of ampicillin, 1 g/L of metronidazole, and 1 g/L of neomycin sulfate) to deplete the gut microbiota. Subsequently, mice in the FMT + LPS and FMT + LPS + HUF-H groups were treated with fecal microbiota collected from donor mice. The suspension of fecal microbiota for the FMT + LPS group was provided by ALI donor mice treated with saline, while that for the FMT + LPS + HUF-H group was provided by ALI donor mice treated with HUF-H; at the same time as the suspensions, 6 g/kg of HUF extract was administered. The NC and LPS groups were administered with saline. Feces from the donor mice were taken randomly, diluted with saline 1:10 (w/v) and vortexed for 5 min; then, 200 μL of the supernatant was taken and administered by rapid gavage. On day 7 of the experiment, 5 mg/kg of LPS was administered to establish the ALI model.

### 2.7 Determination of the wet-to-dry ratio for the lungs and lung/spleen indices

In accordance with established procedures (Matute-Bello et al., 2011; Rao et al., 2022), lung tissue was collected from experimental mice, weighed, recorded as wet weight, and then heated in an oven at 80 °C for 48 h until a constant weight was reached. The samples were then re-weighed and recorded as dry weight; then, the wet/dry (W/D) ratio was calculated. In addition, the lung and spleen tissues were separated and weighed, and indices were calculated as follows: lung index = lung weight/body weight × 100% and spleen index = spleen weight/body weight × 100%.

### 2.8 Histopathological analysis

To observe histopathological damage, lung and colon tissues were fixed with 4% paraformaldehyde, embedded in paraffin, sectioned with a 5-μm thickness, subjected to hematoxylin and eosin (HE) staining, observed by light microscopy and photographed for analysis. Two professional pathologists used a blinded method to score pathological sections with reference to the following criteria. For lung tissue, scoring was performed according to four factors: alveolar vascular congestion; inflammatory infiltration; alveolar wall thickening and the degree of destruction of the lung structure. A score of 0 to 4 was assigned according to the degree of damage (no damage, slight, mild, moderate or severe); these scores were summated and divided by four to obtain a pathology score.

### 2.9 Immunohistochemical and immunofluorescence analysis

Immunohistochemistry and immunofluorescence analyses were performed according to previously established methods (Liao et al., 2022a). The protein expression of colonic zonula occludens-1 (ZO-1), lung c-Jun N-terminal kinase (JNK), extracellular regulated protein kinase (ERK) and P38MAPK proteins was determined (lot #21773-1-AP, #66210-1-Ig, #11257-1-AP, #14064-1-AP, Proteintech Group, China), and the proportion of positive expression zones was determined by Image-Pro Plus 6.0 software. Positive immunohistochemical expression was shown as brown staining, whereas positive expression of the P38MAPK protein was indicated by red staining.

### 2.10 Measurement of inflammatory mediators

A portion of lung tissue was weighed and mixed with nine volumes of phosphate-buffered saline. Then, the tissue was homogenized with an automatic homogenizer. The supernatant was collected after centrifugation at 3,000 *g* for 20 min. The concentrations of inflammatory factors (TNF-α, IL-1β, IL-6, and IL-17) in the supernatant were then determined by enzyme-linked immunosorbent assays (ELISA) kits (FanKew, Shanghai FANKEL Industrial Co., Ltd., China) in accordance with the manufacturer’s instructions.

### 2.11 Real-time polymerase chain reaction (RT‒qPCR)

Total RNA was extracted from lung tissue samples, and reverse transcribed into cDNA to form a template. qPCR was then performed in a special PCR plate and the AceQ qPCR SYBR Green Master Mix Kit (Vazyme, China). The cycle threshold (Ct) of each sample during PCR was analyzed, and the results were calculated by the 2^−ΔΔCT^ method to determine the expression of each target gene. The target genes were *Serpinb2*, *Fez1*, *Wt1*, *Slc26a4, JNK, ERK and P38MAPK*, and the primer sequences are shown in SupplementaryTable 3.

### 2.12 Transcriptome sequencing

An appropriate amount of mouse lung tissue was collected, and total RNA was extracted, purified and quantified, control total RNA with a total amount of ≥1 μg. RNA integrity was assessed with an Agilent 2,100 Bioanalyzer RNA 6000 Nano Kit 5,067–1,511 (Agilent Technologies Inc., California, United States) and library quality testing was performed with an Agilent 2,100 Bioanalyzer. Next, the raw sequencing results were filtered to remove connector contamination and low-quality reads and to obtain clean data. The reference genome and gene model annotation files were then downloaded from the genome website, and high-quality sequences were aligned to the reference genome with HISAT2 v2.2.1 software. HTSeq v0.9.1 software was then used to count the number of reads on each gene to determine gene expression level. Samples were then analyzed with DESeq v1.38.3 software for differential expression with the following filtering conditions: |log2FoldChange| > 1 and p < 0.05. Kyoto Encyclopedia of Genes and Genomes (KEGG) pathway enrichment analysis was performed with clusterProfiler v4.6.0 software, and gene set enrichment analysis was performed with gene set enrichment analysis (GSEA) v4.1.0 software.

### 2.13 16S rRNA gene sequencing

Total fecal genomic DNA was extracted with a DNeasy PowerSoil Kit DNA Kit from QIAGEN, and the extracted DNA was assayed. The V3-V4 region of bacterial 16S rRNA was subsequently amplified by PCR (forward primer 338 F (5′-ACT​CCT​ACG​GGG​AGG​CAG​CA-3′) and reverse primer 806 R (5′-GGACTACHVGGGTWTCTAAT-3′)). High-throughput sequencing was performed on the Illumina MiSeq platform. The average sequencing depth was 96,270 ± 11,548 reads per sample, and the Q30 score exceeded 95%. Taxonomy was assigned using the SILVA v138 and Greengenes reference database. Amplicon sequence variants were defined using the DADA2 pipeline and classified into seven taxonomic ranks: phylum, class, order, family, genus, and species. Principal component analysis of the dispersion trends across different groups. After sequencing, the sequence data were read with QIIME2 software to determine the microbial diversity index and perform community composition analysis.

### 2.14 Determination of SCFAs

SCFAs were quantified using a standard curve method (Han et al., 2018). Mixed stock solutions of acetate, propionate, and butyrate were prepared in water and serially diluted to construct calibration curves (range: 0.1–500 μg/mL). All curves showed an *R* ^2^ value >0.995. The limits of detection and quantification were 0.05 μg/mL and 0.1 μg/mL, respectively. For sample preparation, an appropriate amount of cecal content was homogenized with water for 1 min and centrifuged at 12,000 rpm for 10 min. The supernatant was mixed with 15% phosphoric acid, and 375 μg/mL of 4-methylpentanoic acid was added as an internal standard. The mixture was then extracted with diethyl ether, and the organic layer was centrifuged at 12,000 rpm for 10 min. Chromatographic analysis was performed on a Thermo Trace 1300 GC system equipped with an Agilent HP-INNOWAX capillary column (30 m × 0.25 mm × 0.25 μm). The injection volume was 1 μL with a split ratio of 10:1; helium carrier gas flow rate was 1.0 mL/min. Mass spectrometry was conducted on a Thermo ISQ 7000 in electron ionization mode with an electron energy of 70 eV, using selected ion monitoring. Matrix effects were evaluated by post-extraction spiking, and recoveries ranged from 85% to 110%.

### 2.15 Network pharmacology

Active components of HUF were screened using the Traditional Chinese Medicine Systems Pharmacology Database and Analysis Platform using specific criteria: an oral bioavailability (OB) ≥ 30% and a drug-likeness (DL) ≥ 0.18. The identified compounds were further filtered through SwissADME to retain compounds that satisfied Lipinski’s rule of five and exhibiting high gastrointestinal absorption; no exceptions were made for key known constituents. The putative targets of the screened compounds were predicted using SwissTargetPrediction. ALI-related targets were retrieved from the GeneCards database. Overlapping targets between compound-predicted targets and disease targets were then identified with a Venn diagram (HiplotPro platform) and considered potential anti-ALI targets. Protein-protein interaction networks were constructed using the STRING database and visualized with Cytoscape 3.7.2. KEGG pathway enrichment analysis was performed using the DAVID database with a significance threshold of p < 0.01. Visualization was carried out using the MicroBioinformatics platform.

### 2.16 Quantification and statistical analysis

Statistical analyses were performed using SPSS 22.0 software. Data are presented as mean ± standard deviation. Normality was assessed using the Shapiro-Wilk test, and the homogeneity of variances was evaluated using Levene’s test. For comparisons among multiple groups, one-way analysis of variance was applied when data met normality and homoscedasticity assumptions, followed by Tukey’s post hoc test. When assumptions were violated, the Kruskal–Wallis test and Dunn’s post hoc test were applied. Each experiment was repeated at least three times per group. A p-value <0.05 was considered statistically significant. Spearman’s correlation analysis is used to assess the relationship between data.

## 3 Results

### 3.1 HPLC analysis of HUF and the identification of chemical components

**FIGURE 1:**
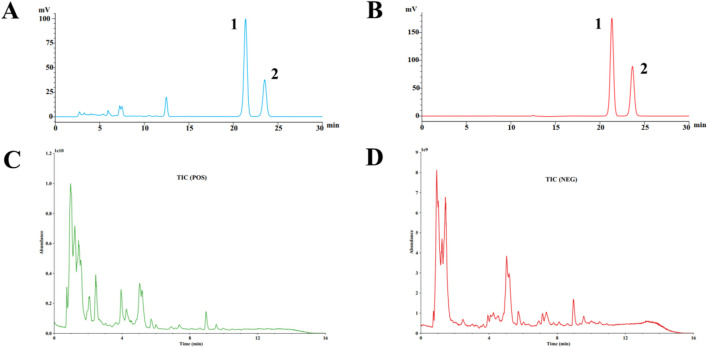
The HPLC chromatogram and TIC of HUF. **(A)** HUF sample solution. 1. Kaempferol. 2. Isorhamnetin. **(B)** Mixed standard solution. 1. Kaempferol. 2. Isorhamnetin. **(C)** Positive ion scanning of HUF samples. **(D)** Negative ion scanning of HUF samples.

The HPLC chromatogram obtained from the QC analysis of HUF is shown in Figure 1A; this demonstrates good separation of the individual components. Using the external standard method, the contents of kaempferol and isorhamnetin were determined to be 2.57 mg/g and 1.37 mg/g, respectively, thus complying with previously established content standards. TIC chromatograms of HUF are shown in Figures 1C,D. Based on MS1/MS2 spectra and comparative analysis against relevant databases, a total of 235 compounds were identified, the majority of which were flavonoids, phenylpropanoids, sugars and glycosides, amino acids, and peptides. A complete list of compounds is provided in Supplementary Table 5.

### 3.2 Pharmacodynamic validation

**FIGURE 2:**
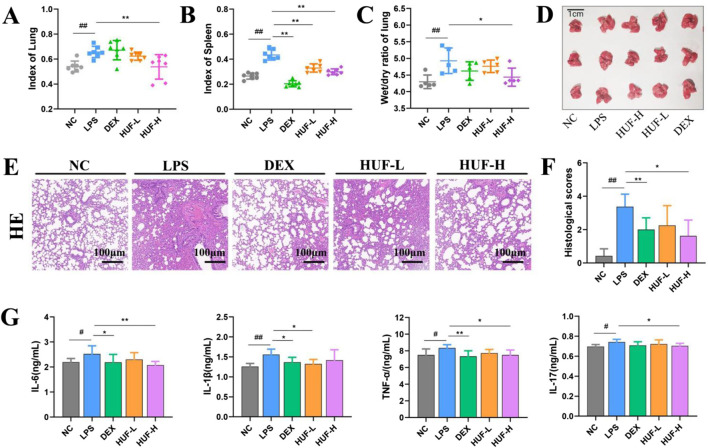
HUF attenuated LPS-induced ALI in mice. **(A)** Lung index. **(B)** Spleen index. **(C)** Wet-to-dry ratio. **(D)** Appearance of each group of lungs. **(E)** Representative images of HE-stained histological sections of the lung. **(F)** Histological scores. **(G)** The levels of IL-6, IL-1β, TNF-α, and IL-17 in the lungs were measured via ELISA. Values were expressed as mean ± SD. ^*^ *p* < 0.05 and ^**^ *p* < 0.01 vs. LPS group; ^#^ *p* < 0.05 and ^##^ *p* < 0.01 vs. NC group.

The lung index was significantly higher in the LPS group than in the NC group. Treatment with HUF-H resulted in a significant reduction of the lung index relative to the LPS group (Figure 2A). The W/D ratio of lung tissue was markedly increased in the LPS group compared with the NC group. HUF-H treatments significantly attenuated this increase, whereas the HUF-L or DEX group showed a modest and nonsignificant reduction (Figure 2C). Representative images of the gross morphology of lung tissue from each group is shown in Figure 2D. The spleen index was significantly higher in LPS-challenged mice than in NC mice. Administration of DEX, HUF-H or HUF-L significantly reduced the spleen index relative to the LPS group (Figure 2B).

HE staining revealed that the NC group exhibited a normal alveolar architecture with thin alveolar septa and no evident inflammatory cell infiltration. In contrast, lung sections from the LPS group exhibited marked pathological alterations, including alveolar wall thickening, severe interstitial edema, hemorrhage, and the extensive infiltration of inflammatory cells. Following DEX and HUF-H treatment, a series of pathological features were significantly alleviated, including alveolar vascular congestion, inflammatory infiltration, and thickening of the alveolar walls (Figure 2E). Histological scoring confirmed a significantly higher injury score in the LPS group than in the NC group. DEX and HUF-H treatments significantly reduced the histological scores relative to the LPS group, while the reduction observed in the HUF-L group was not statistically significant (Figure 2F).

As shown in Figure 2G, the levels of TNF-α, IL-1β, IL-6, and IL-17 in the LPS group were significantly higher than those in the NC group. Compared with the LPS group, DEX treatment significantly suppressed the elevation of TNF-α, IL-1β, and IL-6. In the HUF-H group, TNF-α, IL-6, and IL-17 levels were significantly lower than those in the LPS group; although IL-1β levels were numerically lower than those in the LPS group, this difference was not statistically significant. In the HUF-L group, IL-1β levels were significantly lower than those in the LPS group, while TNF-α, IL-6, and IL-17 levels showed no statistically significant changes compared with the LPS group.

### 3.3 Effects of HUF on the gut microbiota in mice

**FIGURE 3:**
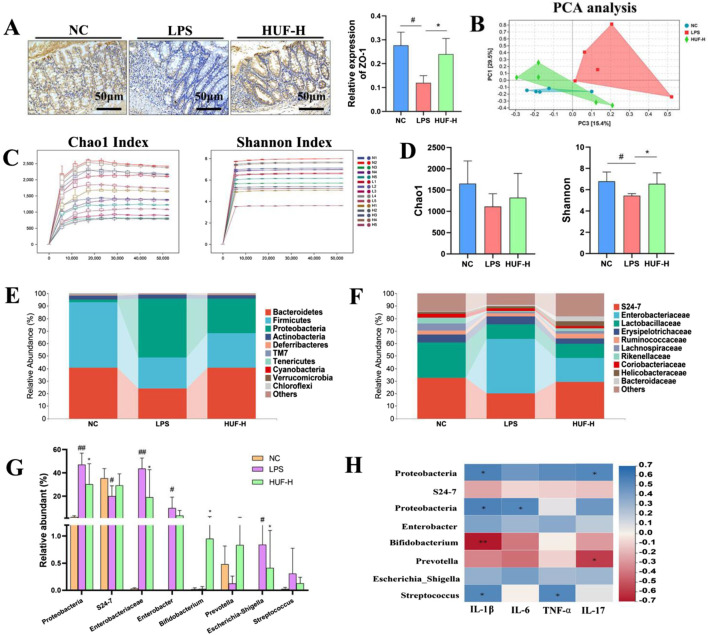
Results of the gut microbiota analysis. **(A)** Representative images of immunohistochemical staining and expression levels of ZO-1 in the gut. **(B)** PCA. **(C)** Chao1 and Shannon index difference tests. **(D)** Analysis of the α diversity of the gut microbiota by Chao1 and Shannon indices. **(E,F)** The relative abundance of the microbial community at the phylum and family levels. **(G)** Comparison of the relative abundances of Proteobacteria, *S24-7, Enterobacteriaceae, Enterobacter, Bifidobacterium, Prevotella, Escherichia-Shigella, and Streptococcus*. **(H)** Correlation analysis of the gut microbiota and environmental factors. Values were expressed as mean ± SD.^*^ *p* < 0.05 and ^**^ *p* < 0.01 vs. LPS group; ^#^ *p* < 0.05 and ^##^ *p* < 0.01 vs. NC group.

Compared with the NC group, the LPS group exhibited a marked reduction in ZO-1 immunoreactivity in colonic tissue. Treatment with HUF-H restored ZO-1 expression to a level comparable to that of the NC group (Figure 3A). Principal component analysis of 16 S rRNA gene sequencing data revealed distinct clustering of gut microbial communities among the NC, LPS, and HUF-H groups. The HUF-H group clustered more closely with the NC group than with the LPS group, indicating a shift in overall microbial community structure following HUF treatment (Figure 3B). Analysis of microbial diversity showed that the Shannon index in the LPS group was significantly lower than that in the NC group, while the Shannon index in the HUF-H group was significantly higher than that in the LPS group (Figure 3D). However, there was no significant difference in the Chao1 index when compared between the three groups. Furthermore, the dilution curves for diversity metrics reached a plateau, indicating that the sequencing depth used was sufficient to ensure the accuracy of the diversity estimates (Figure 3C).

In addition, significant changes in the abundance of various bacterial groups were observed across different taxonomic levels in all groups (Figures 3E,F). At the phylum level, the relative abundance of Proteobacteria was significantly increased in the LPS group relative to the NC group. HUF-H treatment significantly reduced the abundance of Proteobacteria compared with the LPS group (Figure 3E,G). At the family level, the LPS group exhibited an elevated relative abundance of Enterobacteriaceae, which was significantly attenuated by HUF-H treatment (Figure 3F,G). Quantitative analysis at the genus level further identified specific bacterial taxa that were differentially abundant among groups. The relative abundances of *Enterobacter*, *Escherichia-Shigella* were significantly higher in the LPS group than in the NC group. HUF-H treatment significantly reduced the abundances of *Escherichia-Shigella* genera compared with the LPS group (Figure 3G). Conversely, the relative abundances of *S24-7* were significantly lower in the LPS group than in the NC group. HUF-H treatment significantly increased the abundances of *Bifidobacterium* relative to the LPS group. HUF-H also increased the abundance of *S24-7* and *Prevotella*, although this increase was not statistically significant. The relative abundance of *Streptococcus* did not differ significantly between the three groups (Figure 3G). Spearman’s correlation analysis further indicated that the bacterial communities altered by HUF were significantly associated with lung injury markers in a mouse model of ALI (Figure 3H).

### 3.4 Effects of HUF on SCFAs in a mouse model of ALI mice

**FIGURE 4:**
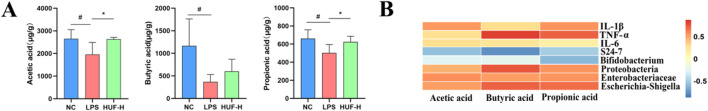
Results of SCFAs sequencing. **(A)** Comparison of the relative abundances of acetic acid, butanoic acid, and propionic acid. **(B)** Correlation analysis of SCFAs and environmental factors. Values were expressed as mean ± SD.^*^ *p* < 0.05 and ^**^ *p* < 0.01 vs. LPS group; ^#^ *p* < 0.05 and ^##^ *p* < 0.01 vs. NC group.

Quantitative measurement of SCFAs revealed that the levels of acetate, propionate, and butyrate in the intestinal contents of LPS-induced mice were significantly reduced, whereas HUF-H treatment effectively increased the concentrations of acetate and propionate (Figure 4A). Further Spearman’s correlation analysis indicated that SCFAs were strongly associated with inflammatory markers of lung injury, positively correlated with the abundance of *Bifidobacterium*, and negatively correlated with the abundance of Proteobacteria (Figure 4B). Collectively, these results suggest that SCFA-related metabolic disorders in ALI mice were significantly associated with disease progression and dysbiosis of the gut microbiota.

### 3.5 FMT treatment confirmed that the effect of HUF on ALI was associated with modulation of the intestinal microenvironment

**FIGURE 5:**
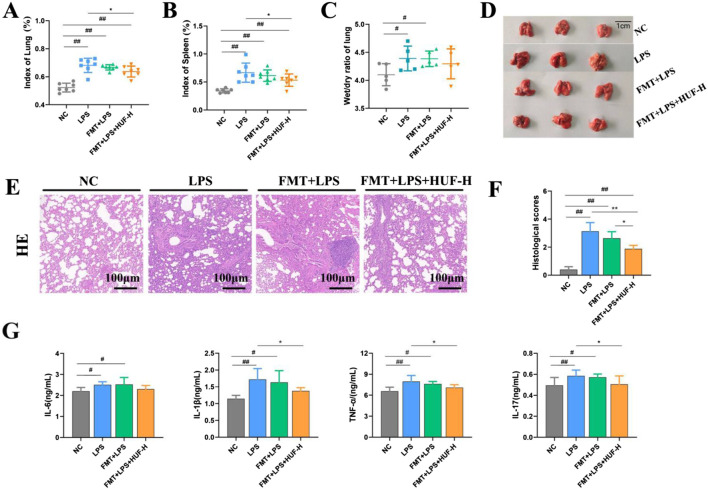
Effects of FMT on disease indicators in recipient mice. **(A)** Lung index. **(B)** Spleen index. **(C)** Wet-to-dry ratio. **(D)** Appearance of each group of lungs. **(E)** Representative images of HE-stained histological sections of the lung. **(F)** Histological scores. **(G)** The levels of IL-6, IL-1β, TNF-α, and IL-17 in the lungs were measured via ELISA. Values were expressed as mean ± SD.^*^ *p* < 0.05 and ^**^ *p* < 0.01 vs. LPS group; ^#^ *p* < 0.05 and ^##^ *p* < 0.01 vs. NC group.

The role of the gut microbiota in the anti-ALI effects of HUF was confirmed by FMT experiments. Compared with the NC group, the LPS group exhibited a significantly elevated lung and spleen index. Mice that received FMT from LPS-treated donors (FMT + LPS group) exhibited lung and spleen indices comparable to that of the LPS group. In contrast, the recipients of FMT from HUF-H-treated donors (FMT + LPS + HUF-H group) exhibited a significantly reduced lung and spleen index relative to the LPS groups (Figure 5A,B). With regards to the W/D ratio, the LPS and FMT + LPS groups were significantly higher than the NC group, although the FMT + LPS + HUF-H group did not show a significant difference when compared to the NC group (Figure 5C). Histopathological analysis of lung tissue sections stained with HE revealed that the LPS group exhibited severe pathological alterations, including thickened alveolar septa, interstitial edema, and extensive infiltration of inflammatory cells (Figure 5E). The FMT + LPS group exhibited a similar degree of histological damage to that of the LPS group. In contrast, lung sections from the FMT + LPS + HUF-H group exhibited marked attenuation of these pathological features. Histological scoring further confirmed that the FMT + LPS + HUF-H group had a significantly lower injury score than the LPS groups (Figure 5F).

Next, we measured the pulmonary concentrations of proinflammatory cytokines by performing ELISA. As shown in Figure 5G, the levels of TNF-α, IL-1β, IL-6, and IL-17 were all significantly elevated in the LPS group compared with the NC group. The FMT + LPS group did not exhibit significant differences in any of the four cytokines compared with the LPS group. In the FMT + LPS + HUF-H group, the levels of TNF-α and IL-17 were significantly lower than those in the LPS group and IL-6 were significantly lower than the FMT + LPS group. The level of IL-1β in the FMT + LPS + HUF-H group was numerically reduced when compared with the LPS and FMT + LPS group, although this difference was not statistically significant.

### 3.6 Transcriptomic effects of HUF on LPS-induced mice

**FIGURE 6:**
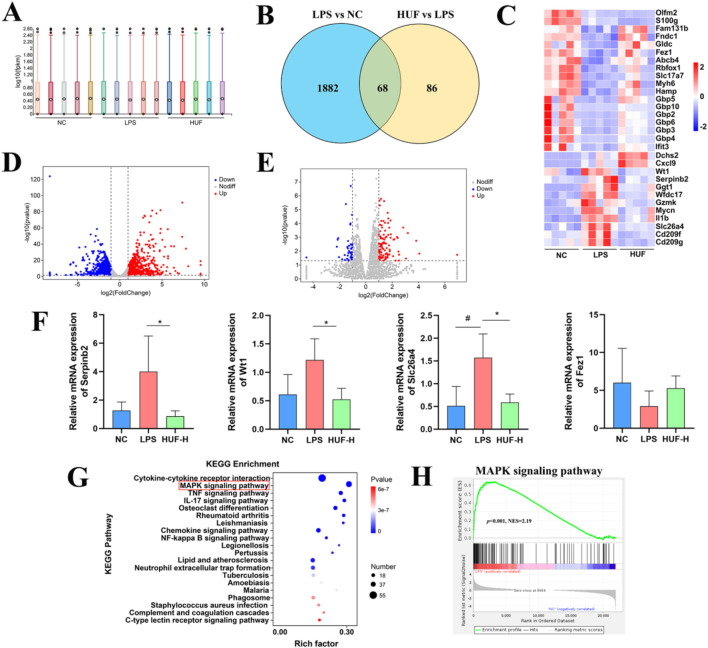
Results of the transcriptome analysis. **(A)** Violin diagram of the FPKM density. **(B)** Venn diagram of the DEGs between LPS vs. NC and HUF vs. LPS. **(C)** Heatmap of the top 30 shared DEGs between LPS vs. NC and HUF vs. LPS. **(D,E)** Volcano plot showing the DEGs in the LPS vs. NC and HUF vs. LPS groups. **(F)** The mRNA expression levels of *Serpinb2, Fez1, Slc26a4*, and *Wt1* in lung tissue were determined via qPCR. **(G)** Enrichment analysis of KEGG pathways associated with the DEGs. **(H)** Results of GSEA of the MAPK signalling pathway. Values were expressed as mean ± SD.^*^ *p* < 0.05 and ^**^ *p* < 0.01 vs. LPS group; ^#^ *p* < 0.05 and ^##^ *p* < 0.01 vs. NC group.

To further elucidate the protective effects of HUF on LPS-induced ALI in mice, we next performed transcriptomic analysis of lung tissues. Fragments per kilobase of exon model per million (FKPN) mapped fragments were shown on violin plots to reflect the level of gene expression in each sample. Analysis revealed that the gene expression levels of all samples were essentially the same, thus verifying that gene expression levels of the same tissues of the same species were similar and that the processing and sequencing results of these samples were reliable (Figure 6A). A comparison of groups by Venn diagrams revealed 1882 differentially expressed genes (DEGs) in the NC group *versus* the LPS group and 86 DEGs in the LPS group *versus* the HUF group, 68 of which were shared genes (Figure 6B). Heatmaps for these DEGs revealed that gene expression was more consistent between the HUF and NC groups than between the HUF and LPS groups (Figure 6C). A volcano map of the DEGs further revealed that 1,086 genes were downregulated after LPS induction while 864 were upregulated (Figure 6D). Moreover, 48 downregulated DEGs and 106 upregulated DEGs were verified after HUF treatment (Figure 6E). Most of the genes for which expression was altered by LPS were restored by HUF treatment, especially *Serpinb2*, *Fez1*, *Wt1* and *Slc26a4*, which presented with large intergroup differences and are closely related to lung disease. Therefore, we further analyzed genes closely related to lung disease by qPCR; analysis revealed that HUF downregulated *Serpinb2*, *Wt1* and *Slc26a4* and upregulated *Fez1*; these findings were consistent with the transcriptome results (Figure 6F). To further investigate the regulatory effects of HUF in ALI mice, KEGG enrichment analyses were performed on the DEGs. KEGG analysis revealed that HUF affected the expression of signaling pathways, such as MAPK, TNF, and IL-17, as well as tuberculosis, pertussis, legionellosis and other respiratory diseases (Figure 6G). GSEA results revealed that LPS induction caused abnormal activation of the MAPK pathway in the lungs (Figure 6H). Collectively, our transcriptomic results demonstrated that HUF intervention in the progression of ALI may be associated with modulation of the expression of key genes, thereby affecting inflammatory processes.

### 3.7 Network pharmacology based on HUF chemical composition

**FIGURE 7:**
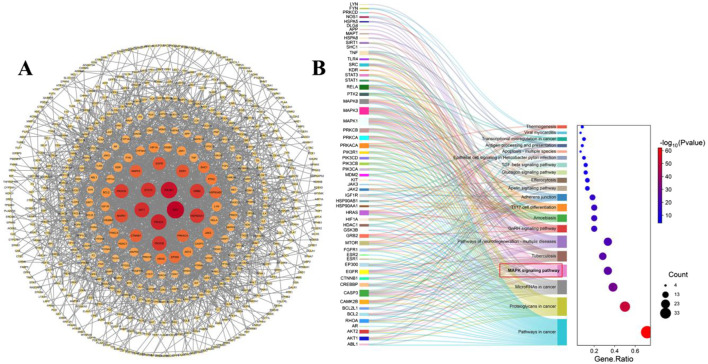
Results of network pharmacology analysis. **(A)** PPI network of core targets. **(B)** KEGG Enrichment analysis bubble plots.

The chemical components of HUF were used to predict the active component targets in the Swiss Target Prediction database. Duplicate targets were removed, resulting in 858 active component targets. By applying the GeneCards and HiplotPro database platforms, we screened for target points intersecting with ALI, imported these into the String database to construct a PPT network, selected strongly associated proteins, and ultimately identified 61 core targets, including SRC, PIK3R1, TNF, PIK3CA, AKT1, STAT3, EGFR, GRB2, and MAPK1 (Figure 7A). KEGG analysis identified a total of 183 pathways; notably, pathways related to cancer, the MAPK signaling pathway, and tuberculosis (Figure 7B).

### 3.8 Validation of key targets

**FIGURE 8:**
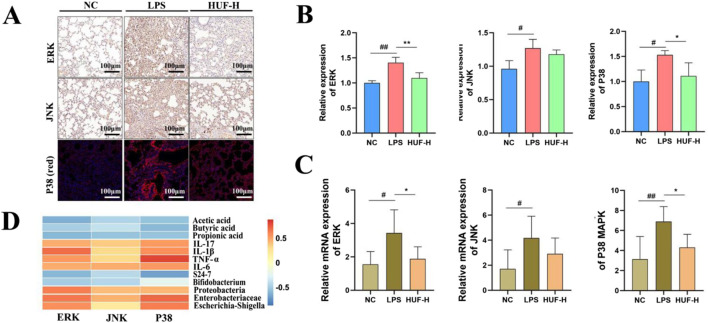
Results of the signalling pathway experiments. **(A)** Immunohistochemical staining of ERK, JNK and immunofluorescence staining of p38 MAPK in lung tissue. **(B)** Relative expression levels of ERK, JNK and P38 MAPK as determined by the area of positive staining. **(C)** mRNA expression levels of *ERK, JNK*, and *P38 MAPK*. **(D)** Correlation analysis of the p38 MAPK pathway and environmental factors. Values were expressed as mean ± SD. *p < 0.05 and **p < 0.01 vs LPS group; ^#^ *p* < 0.05 and ^##^ *p* <0.01 vs NC group.

Based on the results of the transcriptomic and network pharmacology analyses, we next investigated the expression of key MAPK pathway components in lung tissue. As shown in Figures 8A,B, the LPS group exhibited significantly increased immunoreactivity for JNK, ERK, and P38 compared with the NC group. Quantitative analysis of positively stained areas confirmed that JNK, ERK, and P38 protein levels were significantly elevated in the LPS group relative to the NC group. Treatment with HUF-H significantly reduced the expression of ERK and P38 compared with the LPS group, whereas JNK exhibited reduced, but nonsignificant reduction.

The mRNA expression levels of *ERK*, *JNK*, and *p38 MAPK* in lung tissue were quantified by RT-qPCR. Consistent with the findings at the protein level, the LPS group exhibited significantly elevated mRNA levels of all three genes compared with the NC group. HUF-H treatment significantly downregulated the mRNA expression of *ERK* and *p38 MAPK* relative to the LPS group (Figure 8C). Similarly, the expression of *JNK* mRNA was reduced, although this reduction was not significant.

Finally, Spearman’s correlation analysis was conducted to investigate associations between components of the p38 MAPK pathway and the gut microbiota and SCFA levels altered by HUF treatment. As shown in Figure 8D, the protein expression levels of ERK, JNK, and p38 MAPK were positively correlated with the relative abundances of Proteobacteria, Enterobacteriaceae, and *Escherichia-Shigella*, and with the proinflammatory cytokine levels (TNF-α, IL-6, IL-1β, and IL-17). Conversely, the expression levels of these MAPK components were negatively correlated with the relative abundances of *S24-7* and *Bifidobacterium*, as well as with the concentrations of the SCFAs acetate, propionate, and butyrate.

## 4 Discussion

In this study, we found that HUF had a substantial protective effect against LPS-induced ALI. First, HUF significantly improved the gut microbiota and metabolites in a mouse model of ALI; it is possible that this improvement in the gut microbiota may be one of the reasons for the therapeutic effect on ALI. Second, network pharmacology analysis indicated that the anti-ALI effects of HUF are associated with multiple targets and pathways, particularly the inflammatory pathway; this was validated using transcriptomics, immunohistochemistry, and RT-PCR. Third, association analysis revealed that inflammatory markers of lung injury were associated with multiple metabolites and the microbiota. Compared with other traditional Chinese medicines or drugs that can be used to treat ALI and can modulate the gut–lung axis, including Fuzheng Jiedu Decoction, resveratrol (Alharris et al., 2022; Chen et al., 2026), or the transplantation of fecal microbiota and various probiotic preparations (Shen et al., 2023; Ni et al., 2026), HUF exhibits certain similar characteristics, such as the ability to increase the levels of SCFAs, modulate the composition of the gut microbiota, and repair the intestinal barrier. However, HUF also has distinct advantages over these other methods. Compared to the direct supplementation of SCFAs, HUF produces a broader anti-inflammatory effect by regulating multiple targets; compared to single-target drugs (such as TLR4 inhibitors), HUF simultaneously influences microorganisms, metabolites, and host immune signals within the gut–lung axis, reflecting the “multicomponent/multitarget” characteristic of traditional Chinese medicine. Compared to some emerging formulations (Song et al., 2025), as a natural plant extract, HUF offers superior food safety and feasibility for clinical translation. Compared to existing methods, the dual use of use of HUF as a medicine and food product makes it more readily accepted by the general public.

Over recent years, there has been a gradual increase in research on the gut–lung axis, and there is an increasing body of evidence that respiratory and intestinal pathologies are closely linked physiologically and pathologically. In the present study, HUF positively affected the gut–lung axis by influencing multiple aspects of gut microecology. First, we observed that HUF treatment restored the expression of ZO-1, a critical tight junction protein, in the colonic epithelium of ALI mice, thereby protecting the integrity of the intestinal barrier. Previous studies have shown that the intestinal barrier maintains the stability of the gut microbiota, ensures the balance of innate immune responses, and is the first line of defense against pathogens (Tsounis et al., 2023). Disruption of the intestinal barrier structure causes the intestinal microbiota to be disturbed by external factors, leading to the induction of pathogenic bacteria (Iacob and Iacob, 2019). These bacteria, and some of their metabolites, may interfere with the immune system through the gut–lung axis, triggering abnormal inflammatory responses (Dickson et al., 2016). Indeed, the loss of ZO-1 expression has been implicated in increased intestinal permeability and the exacerbation of systemic inflammation in various models of disease (Kuo et al., 2021). We demonstrated that HUF upregulated ZO-1 expression; this suggests that reinforcement of the intestinal physical barrier may represent one of the mechanisms by which HUF limits the dissemination of gut-derived inflammatory mediators to the lungs. This interpretation was further supported by the reduction in pulmonary inflammatory markers observed in HUF-treated mice. Future studies employing intestinal permeability assays (e.g., fluorescein isothiocyanate-dextran) would help to functionally validate this protective barrier effect (Bao et al., 2023). Second, this structural maintenance likely contributes to a more resilient microbial ecosystem in the gut, thus reducing susceptibility to dysbiosis (Kuo et al., 2021). It is important to note, however, that the increased microbial diversity observed following HUF treatment does not directly equate to physical thickening of the epithelial barrier. Rather, the beneficial effects on barrier function are likely to be mediated indirectly through microbial metabolites such as SCFAs, which are known to promote the expression of tight junction proteins and enhance mucosal defense mechanisms. Indeed, butyrate has been shown to upregulate the expression of ZO-1 and occludin via the activation of AMP-activated protein kinase and the inhibition of histone deacetylases, thereby fortifying the intestinal barrier (Bordin et al., 2004; Peng et al., 2009).

Next, we analyzed and identified the target microbiota through which HUF improves ALI through the gut–lung axis. At the genus level, HUF reduced the abundance of Proteobacteria. In some lung diseases, an increased abundance of intestinal Proteobacteria has become a notable marker of disease (Rizzatti et al., 2017; Liao et al., 2023). At the species level, the relative abundance of *S24-7* increased significantly in response to HUF. Previous studies have shown that *S24-7* can regulate the MAPK pathway, restore the intestinal microbiota, reduce inflammatory cell infiltration, weaken the activity of tumor necrosis activity, and inhibit inflammatory responses (Zou et al., 2020; Gao et al., 2025). At the genus level, some beneficial bacteria also served as marker species for the HUF group. For example, *Bifidobacterium* has been shown to improve lung damage in mice by regulating the number of inflammatory cells, thereby ensuring the health of the intestinal and lung tissues (Nan et al., 2023). Moreover, some pathogenic bacteria, such as *Enterobacter* and *Escherichia-Shigella*, were downregulated after HUF treatment. It has been reported that the abnormal elevation of *Escherichia-Shigella* is an important marker for various lung diseases (Buendía et al., 2018); furthermore, *Enterobacter* is one of the key factors in bacterial pneumonia (Rajpal et al., 2023). Further correlation analysis revealed that the gut microbiota regulated by HUF was significantly associated with indicators of ALI. These findings confirm that the regulatory effect of HUF on the gut microbiota may be important for improving ALI.

We also determined that the regulatory mechanism of HUF involves the metabolism of SCFAs. SCFAs derived from the gut microbiota can influence lung health through the gut–lung axis, and that the supplementation of SCFAs can reduce inflammation, oxidative stress, and metabolic changes in the lungs of a mouse model of ALI (Hildebrand et al., 2023). As a representative SCFA, acetate can inhibit the expression levels of the inflammatory factors IL-6 and TNF-α in the lung tissue of a mouse model of ALI by reducing the expression of NF-κB (Hung et al., 2022). In this study, HUF increased the levels of SCFAs, such as acetate and propionate, which may provide an opportunity to improve the lung environment. Spearman’s correlation analysis revealed a significant correlation between SCFAs and the gut microbiota, thus establishing a mechanistic link between the two.

The effects of HUF on the gut microbiota and its metabolites were significant; however, whether this regulatory effect is one of the direct causes by which HUF improves symptoms in a mouse model of ALI remains unclear. To address this, our research group conducted preliminary validation via FMT experiments. Analysis revealed that the administration of a fecal microbiota mixture with LPS failed to alleviate the disease symptoms of pseudogerm-free ALI mice, whereas the administration of a fecal microbiota mixture with HUF alleviated the related symptoms of pseudogerm-free ALI mice, thus suggesting that the regulatory effect of HUF on the gut microbiota and its metabolites may be one of the reasons underlying improvements in the symptoms related to ALI. When compared with the LPS group, direct HUF-H treatment reduced the lung index, spleen index, and W/D ratio by 18%, 31%, and 10%, respectively, whereas FMT + LPS + HUF-H reduced these parameters by 7%, 20%, and 2%, respectively. Similarly, IL-6 levels decreased by 18% in the HUF-H group and by 8% in the FMT + LPS + HUF-H group. Collectively, these results indicate that the protective effect of FMT was limited, providing only partial protection, thus suggesting that the mechanism of action underling the effect of HUF is microbiota-dependent and microbiota-independent.

Transcriptomic technology has often be used to investigate the complexity of the multiple components and multiple targets of herbal medicines. In the present study, analysis of lung RNA-seq data revealed that under the influence of multiple factors, the expression levels of GBP family genes significantly associated with anti-inflammatory effects and immune regulation, such as *GBP2*, *GBP3*, and *GBP5*, were significantly reduced under LPS induction (Sun et al., 2025). HUF, however, can restore the expression levels of this family of genes. In addition, the activity of the *Il1β* gene was attenuated by HUF treatment. As an important ligand for the inflammatory factor IL-1β, *Il1β* plays an initiating role in the activation of inflammatory signals, such as the MAPK and TNF pathways, and is considered an important player in airway inflammation (Yi et al., 2018). Further qPCR analysis revealed that some of the differentially expressed genes may represent candidate genes for HUF functionality. For example, *SerpinB2* is known to be significantly upregulated under multiple inflammatory conditions, and the high expression levels of this gene in activated macrophages is associated with a variety of human inflammatory diseases (Schroder et al., 2011). *Slc26a4*, on the other hand, may be involved in airway inflammation and epithelial changes via the activation of inflammatory vesicles and the induction of IL-17 and IFN-γ (Lee et al., 2020). *Wt1* has been positively associated with lung cancer (Wang et al., 2013), and *Fez1* has been negatively correlated with lung cancer (Nonaka et al., 2005). Therefore, HUF is likely to improve ALI by regulating gene networks associated with lung inflammation.

It is likely that HUF exerts a direct effect on lung tissue, as its anti-inflammatory effects and ability to regulate the p38 MAPK pathway were directly observed in our transcriptomic and immunohistochemical studies of lung tissue from mice that did not receive FMT. Furthermore, our previous research, as well as a prior study on HUF polysaccharides, confirmed the significant anti-inflammatory and antioxidant potential of HUF (Liao et al., 2022b; Li et al., 2023). Compounds in HUF, such as kaempferol, have been shown to exhibit direct anti-inflammatory and antioxidant effects and are capable of protecting lung epithelial cells from damage (Li et al., 2025).

In this study, transcriptomics and network pharmacology results predicted that the MAPK pathway is highly correlated with the ameliorative effects of HUF on ALI. MAPK often serves as an upstream signal in inflammatory cascades, regulating ERK, JNK, and p38 MAPK, which collectively promote the infiltration of inflammatory cells, disrupt the alveolar–capillary barrier, cause pulmonary edema, and exacerbate ALI (Saleem, 2024; Zheng et al., 2024). Our quantitative analyses further demonstrated that HUF treatment reduced the expression of these MAPK branches at the protein and mRNA levels. Specifically, compared with the LPS group, HUF reduced the immunohistochemical staining of p38 by 27.45%, for ERK by 21.85%, and for JNK by only 7.35%. At the mRNA level, these reductions were 37.49% for p38, 45.10% for ERK, and 30.54% for JNK. Thus, p38 and ERK were the most robustly modulated MAPK branches, whereas JNK showed relatively minor changes. Previous studies have consistently linked the MAPK pathway to ALI, although few have connected the MAPK to the gut–lung axis. It is evident that there is a close bidirectional relationship between gut microbiota/metabolites and MAPK signaling. For example, butyrate can reach pulmonary immune cells via the gut–lung axis and alleviate asthma by inhibiting p38 MAPK (Chen et al., 2021; Shao et al., 2024; Yu et al., 2025). Conversely, overactivated p38 MAPK has been shown to induce gut epithelial cells to release proinflammatory factors, disrupting integrity of the gut barrier and promoting dysbiosis (Zhang et al., 2021; Liu et al., 2022; Van Gerrewey and Chung, 2024). Our analyses confirmed that HUF suppresses MAPK activation (especially p38 and ERK) in the lungs, while Spearman’s correlation analysis revealed that changes in these MAPK branches were significantly correlated with alterations in the gut microbiota and SCFAs. Although SCFA levels correlated with parameters of lung inflammation, we did not perform direct supplementation (e.g., acetate or butyrate) in this study. Therefore, the proposed SCFA-MAPK-lung inflammation axis remains correlative and inferential at this stage.

There are several limitations to this study that need to be considered. First, only male C57BL/6 mice were used. Second, only a single model of ALI was tested. Third, in the FMT experiments, the group treated with a combination of antibiotics alone was not evaluated. Fourth, we did not conduct direct validation experiments involving SCFA supplementation. Finally, we did not apply Western blotting to further validate the protein pathways identified. Future research needs to consider a greater number of experimental components to consolidate our current findings and continue to investigate the pharmacological mechanisms of HUF with advance technologies.

## 5 Conclusion

Our analyses suggest that HUF is associated with the attenuation of ALI, and achieves this effect by modulating the gut–lung axis and inflammatory pathways. In China, HUF is currently classified as a traditional Chinese medicinal herb and a functional food resource. Furthermore, HUF is included in some local pharmacopoeias and is primarily utilized in the form of a decoction for the treatment of certain respiratory diseases. To date, there have been no reports of HUF causing serious adverse effects in humans. However, before conducting clinical trials, it is necessary to prepare a standardized extract with consistent quality across batches and to conduct thorough toxicity testing. In summary, the above results indicate that HUF holds promising prospects for development; this study provides experimental data that can serve as a reference for advancing HUF into clinical trials (Fu et al., 2014; Xu et al., 2018).

## Data Availability

The original contributions presented in the study are included in the article/Supplementary Material, further inquiries can be directed to the corresponding author. The data presentedin the study are deposited in the https://www.ncbi.nlm.nih.gov/sra/PRJNA1471994 repository, accession number PRJNA1471994.

## References

[B1] Alharris E. Mohammed A. Alghetaa H. Zhou J. Nagarkatti M. Nagarkatti P. (2022). The ability of resveratrol to attenuate ovalbumin-mediated allergic asthma is associated with changes in microbiota involving the gut-lung axis, enhanced barrier function and decreased inflammation in the lungs. Front. Immunol. 13, 805770. 10.3389/fimmu.2022.805770 35265071 PMC8898895

[B2] Bao K. Wang M. Liu L. Zhang D. Jin C. Zhang J. (2023). Jinhong decoction protects sepsis-associated acute lung injury by reducing intestinal bacterial translocation and improving gut microbial homeostasis. Front. Pharmacol. 14, 1079482. 10.3389/fphar.2023.1079482 37081964 PMC10110981

[B3] Bordin M. D'Atri F. Guillemot L. Citi S. (2004). Histone deacetylase inhibitors up-regulate the expression of tight junction proteins. Mol. Cancer Res. 2 (12), 692–701. 15634758

[B4] Budden K. F. Gellatly S. L. Wood D. L. Cooper M. A. Morrison M. Hugenholtz P. (2017). Emerging pathogenic links between microbiota and the gut-lung axis. Nat. Rev. Microbiol. 15 (1), 55–63. 10.1038/nrmicro.2016.142 27694885

[B5] Buendía E. Zakzuk J. San-Juan-Vergara H. Zurek E. Ajami N. J. Caraballo L. (2018). Gut microbiota components are associated with fixed airway obstruction in asthmatic patients living in the tropics. Sci. Rep. 8 (1), 9582. 10.1038/s41598-018-27964-3 29941875 PMC6018556

[B6] Chen Z. Y. Xiao H. W. Dong J. L. Li Y. Wang B. Fan S. J. (2021). Gut microbiota-derived PGF2α fights against radiation-induced lung toxicity through the MAPK/NF-κB pathway. Antioxidants (Basel) 11 (1), 65. 10.3390/antiox11010065 35052569 PMC8773112

[B7] Chen J. Pan S. Huo W. Wang W. Tan Z. Wu Y. (2026). Fuzheng jiedu formula ameliorates acute lung injury by modulating Gut microbiota to enhance short-chain fatty acid. J. Inflamm. Res. 19, 5–20. 10.2147/JIR.S556752 41709968 PMC12912167

[B8] Chioma O. S. Mallott E. K. Chapman A. Van Amburg J. C. Wu H. Shah-Gandhi B. (2022). Gut microbiota modulates lung fibrosis severity following acute lung injury in mice. Commun. Biol. 5 (1), 1401. 10.1038/s42003-022-04357-x 36543914 PMC9772329

[B9] Dickson R. P. Singer B. H. Newstead M. W. Falkowski N. R. Erb-Downward J. R. Standiford T. J. (2016). Enrichment of the lung microbiome with gut bacteria in sepsis and the acute respiratory distress syndrome. Nat. Microbiol. 1 (10), 16113. 10.1038/nmicrobiol.2016.113 27670109 PMC5076472

[B10] Dong Y. He L. Zhu Z. Yang F. Ma Q. Zhang Y. (2024). The mechanism of gut-lung axis in pulmonary fibrosis. Front. Cell Infect. Microbiol. 14, 1258246. 10.3389/fcimb.2024.1258246 38362497 PMC10867257

[B38] Fu S. Huang X. Wu L. Long F. Wei X. Jiang W. (2014). Experimental study on the antitussive, apophlegmatic and antiasthmatic effect of extract of jianhua. Med. Plant 5 (2), 31–33.

[B11] Gao F. Wang F. Wang D. Du G. (2025). Bibliometric analysis of the S24-7 family and its association with health. Front. Microbiol. 16, 1571883. 10.3389/fmicb.2025.1571883 40406341 PMC12095373

[B12] Han X. Guo J. You Y. Yin M. Ren C. Zhan J. (2018). A fast and accurate way to determine short chain fatty acids in mouse feces based on GC-MS. J. Chromatogr. B Anal. Technol. Biomed. Life Sci. 1099, 73–82. 10.1016/j.jchromb.2018.09.013 30243116

[B13] Hildebrand C. B. Lichatz R. Pich A. Mühlfeld C. Woltemate S. Vital M. (2023). Short-chain fatty acids improve inflamm-aging and acute lung injury in old mice. Am. J. Physiol. Lung Cell Mol. Physiol. 324 (4), L480–L492. 10.1152/ajplung.00296.2022 36802219

[B14] Hughes K. T. Beasley M. B. (2017). Pulmonary manifestations of Acute lung injury: more than just diffuse alveolar damage. Arch. Pathol. Lab. Med. 141 (7), 916–922. 10.5858/arpa.2016-0342-RA 27652982

[B15] Hung K. Y. Wu S. Y. Pao H. P. Liao W. I. Chu S. J. (2022). Acetate, a gut bacterial product, ameliorates ischaemia‒reperfusion induced acute lung injury in rats. Int. Immunopharmacol. 111, 109136. 10.1016/j.intimp.2022.109136 35964409

[B16] Iacob S. Iacob D. G. (2019). Infectious threats, the intestinal barrier, and its trojan horse: dysbiosis. Front. Microbiol. 10, 1676. 10.3389/fmicb.2019.01676 31447793 PMC6692454

[B17] Kabel A. M. Omar M. S. Elmaaboud M. A. A. (2016). Amelioration of bleomycin-induced lung fibrosis in rats by valproic acid and butyrate: role of nuclear factor kappa-B, proinflammatory cytokines and oxidative stress. Int. Immunopharmacol. 39, 335–342. 10.1016/j.intimp.2016.08.008 27526269

[B18] Kuo W. T. Zuo L. Odenwald M. A. Madha S. Singh G. Gurniak C. B. (2021). The tight junction protein ZO-1 is dispensable for barrier function but critical for effective mucosal repair. Gastroenterology 161 (6), 1924–1939. 10.1053/j.gastro.2021.08.047 34478742 PMC8605999

[B19] Lee J. U. Lee H. J. Kim J. N. Kim M. K. Kim S. R. Chang H. S. (2020). Effects of ammonium chloride on ozone-induced airway inflammation: the role of Slc26a4 in the lungs of mice. J. Korean Med. Sci. 35 (32), e272. 10.3346/jkms.2020.35.e272 32808511 PMC7431289

[B20] Li C. Zhang Y. Zhao C. Fu X. (2023). Physicochemical characterization, antioxidative and immunoregulatory activity of polysaccharides from the flower of Hylocereus undatus (Haw.) Britton et Rose. Int. J. Biol. Macromol. 251, 126408. 10.1016/j.ijbiomac.2023.126408 37598818

[B21] Li S. Wang S. Zhang L. Ka Y. Zhou M. Wang Y. (2025). Research progress on pharmacokinetics, anti-inflammatory and immunomodulatory effects of kaempferol. Int. Immunopharmacol. 152, 114387. 10.1016/j.intimp.2025.114387 40054326

[B22] Liao W. Liu W. Yan Y. Li L. Tong J. Huang Y. (2022a). Hylocereus undatus flower extract suppresses OVA-Induced allergic asthma in BALb/c mice by reducing airway inflammation and modulating gut microbiota. Biomed. Pharmacother. 153, 113476. 10.1016/j.biopha.2022.113476 35977054

[B23] Liao W. Liu W. Zhou X. Chen X. Huang Y. Jiang W. (2022b). Anticough, expectorant, anti-inflammatory, analgesic effects and preliminary mechanism of the extract from Hylocereus undatus flowers. SHi Zhen Chin. Med. 33 (04), 821–823.

[B24] Liao W. Jiang W. Liu W. Zhou X. Q. Liu X. Fu S. J. (2022c). Study on quality standard of bawanghua medicinal material. China Pharm. 33 (14), 1736–1741.

[B25] Liao W. Wu F. Pang L. He B. Tong J. Qin J. (2023). Hylocereus undatus flower suppresses DSS-Induced colitis in mice by reducing intestinal inflammation, repairing the intestinal physical barrier, and modulating gut and lung microbiota. J. Funct. Foods. 110, 105820. 10.1016/j.jff.2023.105820

[B26] Liu L. Liu Y. Guo X. Jin X. Yan W. Lin B. (2022). Activation of p38 mitogen-activated protein kinase pathway by lipopolysaccharide aggravates postoperative ileus in colorectal cancer patients. J. Gastroenterol. Hepatol. 37 (3), 518–530. 10.1111/jgh.15760 34907602

[B658] Matute-Bello G. Downey G. Moore B. B. Groshong S. D. Matthay M. A. Slutsky A. S. (2011). An official American Thoracic Society workshop report: features and measurements of experimental acute lung injury in animals. Am. J. Respir. Cell Mol. Biol. 44 (5), 725–738. 10.1165/rcmb.2009-0210ST 21531958 PMC7328339

[B27] Nan X. Zhao W. Liu W. H. Li Y. Li N. Hong Y. (2023). Bifidobacterium animalis subsp. lactis BL-99 ameliorates colitis-related lung injury in mice by modulating short-chain fatty acid production and inflammatory monocytes/macrophages. Food Funct. 14 (2), 1099–1112. 10.1039/d2fo03374g 36594489

[B28] Ni J. Shen J. Wang F. Wu Y. Qiu B. Zhou Z. (2026). Lactobacillus fermentum remodeled the lung microbiota by crosstalk with the gut and lungs and regulated the PI3K-AKT pathway to alleviate acute lung injury. Food Funct. 17 (2), 991–1006. 10.1039/d5fo04619j 41504299

[B29] Nonaka D. Fabbri A. Roz L. Mariani L. Vecchione A. Moore G. W. (2005). Reduced FEZ1/LZTS1 expression and outcome prediction in lung cancer. Cancer Res. 65 (4), 1207–1212. 10.1158/0008-5472.CAN-04-3461 15735004

[B30] Peng L. Li Z. R. Green R. S. Holzman I. R. Lin J. (2009). Butyrate enhances the intestinal barrier by facilitating tight junction assembly *via* activation of AMP-Activated protein kinase in Caco-2 cell monolayers. J. Nutr. 139 (9), 1619–1625. 10.3945/jn.109.104638 19625695 PMC2728689

[B31] Rajpal K. Kumar S. Saurabh K. Kumari N. Kumar R. Kumar R. (2023). Post-COVID-19 cavitary lung lesion due to Aspergillus flavus and *Enterobacter cloacae* in a patient suffering from COVID-19 pneumonia - a case report. Access Microbiol. 5 (6), acmi000457. 10.1099/acmi.0.000457 37424552 PMC10323781

[B32] Rao Z. Zeng J. Li X. Peng L. Wang B. Luan F. (2022). JFNE-A isolated from jing-fang n-butanol extract attenuates lipopolysaccharide-induced acute lung injury by inhibiting oxidative stress and the NF-κB signaling pathway *via* promotion of autophagy. Phytomedicine 96, 153891. 10.1016/j.phymed.2021.153891 35026506

[B33] Rizzatti G. Lopetuso L. R. Gibiino G. Binda C. Gasbarrini A. (2017). Proteobacteria: a common factor in human diseases. Biomed. Res. Int. 2017, 9351507. 10.1155/2017/9351507 29230419 PMC5688358

[B34] Saleem S. (2024). Targeting MAPK signaling: a promising approach for treating inflammatory lung disease. Pathol. Res. Pract. 254, 155122. 10.1016/j.prp.2024.155122 38246034

[B35] Schroder W. A. Major L. Suhrbier A. (2011). The role of SerpinB2 in immunity. Crit. Rev. Immunol. 31 (1), 15–30. 10.1615/critrevimmunol.v31.i1.20 21395508

[B36] Shao R. Tan X. Pan M. Huang J. Huang L. Bi B. (2024). Inulin alters gut microbiota to alleviate post-stroke depressive-like behavior associated with the IGF-1-mediated MAPK signaling pathway. Brain Behav. 14 (1), e3387. 10.1002/brb3.3387 38376033 PMC10794126

[B37] Shen J. Wang S. Xia H. Han S. Wang Q. Wu Z. (2023). Akkermansia muciniphila attenuated lipopolysaccharide-induced acute lung injury by modulating the gut microbiota and SCFAs in mice. Food Funct. 14 (23), 10401–10417. 10.1039/d3fo04051h 37955584

[B39] Song H. Ma Y. Peng L. Gao F. Fan X. Yang M. (2025). Platinum-doped emodin carbon dots mitigate sepsis-induced lung injury by targeting the gut-lung axis. J. Nanobiotechnology 24 (1), 84. 10.1186/s12951-025-03972-0 41454342 PMC12849688

[B40] Sun X. Jin G. Zhou H. Wang Y. Dai F. Zhou G. (2025). Role of guanylate-binding protein 5 in inflammatory diseases, immune diseases, cancers, and its potential therapeutic implications. Inflammopharmacology 33 (5), 2217–2229. 10.1007/s10787-025-01727-9 40192997

[B41] Tang J. Xu L. Zeng Y. Gong F. (2021). Effect of gut microbiota on LPS-Induced acute lung injury by regulating the TLR4/NF-kB signaling pathway. Int. Immunopharmacol. 91, 107272. 10.1016/j.intimp.2020.107272 33360370

[B42] Tsounis E. P. Triantos C. Konstantakis C. Marangos M. Assimakopoulos S. F. (2023). Intestinal barrier dysfunction as a key driver of severe COVID-19. World J. Virol. 12 (2), 68–90. 10.5501/wjv.v12.i2.68 37033148 PMC10075050

[B43] Van Gerrewey T. Chung H. S. (2024). MAPK cascades in plant Microbiota structure and functioning. J. Microbiol. 62 (3), 231–248. 10.1007/s12275-024-00114-3 38587594

[B44] Verma A. Bhagchandani T. Rai A. Nikita Sardarni U. K. Bhavesh N. S. (2024). Short-chain fatty acid (SCFA) as a connecting link between microbiota and gut-lung Axis-A potential therapeutic intervention to improve lung health. ACS Omega 9 (13), 14648–14671. 10.1021/acsomega.3c05846 38585101 PMC10993281

[B45] Wang X. Gao P. Lin F. Long M. Weng Y. Ouyang Y. (2013). Wilms' tumour suppressor gene 1 (WT1) is involved in the carcinogenesis of Lung cancer through interaction with PI3K/Akt pathway. Cancer Cell Int. 13 (1), 114. 10.1186/1475-2867-13-114 24228711 PMC3833182

[B46] Wang B. Lin Y. Zhou M. Fu S. Zhu B. Chen Y. (2022). Polysaccharides from Tetrastigma Hemsleyanum Diels et Gilg attenuate LPS-induced acute lung injury by modulating TLR4/COX-2/NF-κB signaling pathway. Biomed. Pharmacother. 155, 113755. 10.1016/j.biopha.2022.113755 36182735

[B47] Xu J. Liu Z. Zhan W. Jiang R. Yang C. Zhan H. (2018). Recombinant TsP53 modulates intestinal epithelial barrier integrity *via* upregulation of ZO-1 in LPS-Induced septic mice. Mol. Med. Rep. 17 (1), 1212–1218. 10.3892/mmr.2017.7946 29115466

[B48] Yan M. Man S. Liang Y. Ma L. Guo L. Huang L. (2023). Diosgenin alleviates nonalcoholic steatohepatitis through affecting liver-gut circulation. Pharmacol. Res. 187, 106621. 10.1016/j.phrs.2022.106621 36535571

[B49] Yang H. H. Duan J. X. Liu S. K. Xiong J. B. Guan X. X. Zhong W. J. (2020). A COX-2/sEH dual inhibitor PTUPB alleviates lipopolysaccharide-induced acute lung injury in mice by inhibiting NLRP3 inflammasome activation. Theranostics 10 (11), 4749–4761. 10.7150/thno.43108 32308747 PMC7163435

[B50] Yi G. Liang M. Li M. Fang X. Liu J. Lai Y. (2018). A large lung gene expression study identifying IL1B as a novel player in airway inflammation in COPD airway epithelial cells. Inflamm. Res. 67 (6), 539–551. 10.1007/s00011-018-1145-8 29616282

[B51] Yu B. Pei C. Peng W. Zheng Y. Fu Y. Wang X. (2025). Microbiota-derived butyrate alleviates asthma *via* inhibiting Tfh13-mediated IgE production. Signal Transduct. Target Ther. 10 (1), 181. 10.1038/s41392-025-02263-2 40473603 PMC12141656

[B52] Zhang Z. Zhang Q. Li F. Xin Y. Duan Z. (2021). Contributions of HO-1-Dependent MAPK to regulating intestinal barrier disruption. Biomol. Ther. Seoul. 29 (2), 175–183. 10.4062/biomolther.2020.112 33093265 PMC7921856

[B53] Zheng B. Li M. Lan E. Ding W. Gao L. Tang Y. (2024). GSK3179106 ameliorates lipopolysaccharide-induced inflammation and acute lung injury by targeting p38 MAPK. Respir. Res. 25 (1), 388. 10.1186/s12931-024-03012-9 39468539 PMC11520791

[B54] Zhou X. Liao Y. (2021). Gut-Lung crosstalk in sepsis-induced acute lung injury. Front. Microbiol. 12, 779620. 10.3389/fmicb.2021.779620 35003009 PMC8733643

[B55] Zhu T. Hu B. Ye C. Hu H. Yin M. Zhang Z. (2022). Bletilla striata oligosaccharides improve ulcerative colitis by regulating gut Microbiota and intestinal metabolites in dextran sulfate sodium-induced mice. Front. Pharmacol. 13, 867525. 10.3389/fphar.2022.867525 35548331 PMC9081565

[B56] Ziaka M. Exadaktylos A. (2024). Gut-derived immune cells and the gut-lung axis in ARDS. Crit. Care 28 (1), 220. 10.1186/s13054-024-05006-x 38965622 PMC11225303

[B57] Zou J. Shen Y. Chen M. Zhang Z. Xiao S. Liu C. (2020). Lizhong decoction ameliorates ulcerative colitis in mice *via* modulating gut microbiota and its metabolites. Appl. Microbiol. Biotechnol. 104 (13), 5999–6012. 10.1007/s00253-020-10665-1 32418127

