# Supplementary material for: Hylocereus undatus flower inhibits lipopolysaccharide-induced acute lung injury in mice by regulating the gut-lung axis and inflammatory response
Source: Front Pharmacol. 2026 Jun 15;17:1803336. doi: 10.3389/fphar.2026.1803336 (PMC13310986; doi:10.3389/fphar.2026.1803336)
Supplement: Supplementary file 1 [file Supplementaryfile1.docx]

Supplementary Material

# Supplementary Figures

#
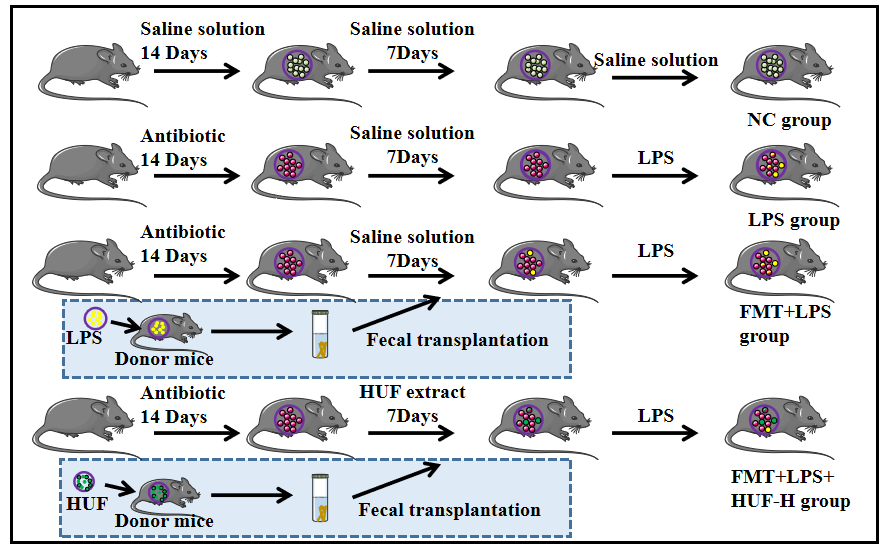


**Supplementary Figure 1.** Demonstration of the FMT Experiment Process.

# Supplementary Tables

**Tab. 1. HPLC elution gradient in the chemical composition analysis of HUF extract**

| Time | A% | B% |
| --- | --- | --- |
| 0.0 | 95.0 | 5.0 |
| 2.0 | 95.0 | 5.0 |
| 4.0 | 70.0 | 30.0 |
| 8.0 | 50.0 | 50.0 |
| 10.0 | 20.0 | 80.0 |
| 14.0 | 0.0 | 100.0 |
| 15.0 | 0.0 | 100.0 |
| 15.1 | 95.0 | 5.0 |
| 16.0 | 95.0 | 5.0 |

**Tab. 2. Mass spectrometry conditions in the chemical composition analysis of HUF extract**

| Parameters | Positive ions | Negative ions |
| --- | --- | --- |
| Spray Voltage (V) | 3800 | -3000 |
| Capillary Temperature (°C) | 320 | 320 |
| Aux gas heater temperature (°C) | 350 | 350 |
| Sheath Gas Flow Rate (Arb) | 35 | 35 |
| Aux gas flow rate (Arb) | 8 | 8 |
| S-lens RF level | 50 | 50 |
| Mass range (m/z) | 100-1500 | 100-1500 |
| Full ms resolution | 70000 | 70000 |
| MS/MS resolution | 17500 | 17500 |
| NCE/stepped NCE | 10,20,40 | 10,20,40 |

**Tab. 3. Primer sequences**

| Gene | Primer sequences (5' to 3 ') |
| --- | --- |
| Actin(nei)-F | TATGGAATCCTGTGGCATC |
| Actin(nei)-R | GTGTTGGCATAGAGGTCTT |
| Slc26a4-F | ATCCTCTCCATTATCTACAC |
| Slc26a4-R | TCCTTAACAGCCATACAG |
| Fez1-F | TGAGTCTGGATGAAGAGTT |
| Fez1-R | AGGTGATGAGGAGATGTG |
| Wt1-F | AGGTTTTCTCGCTCAGACCA |
| Wt1-R | CCTGGTGTGGGTCTTCAGAT |
| Serpinb2-F | TGTTCCTCCATGCAAAGCTG |
| Serpinb2-R | CGTCCTCAATCTCATCGGGA |
| p38 MAPK | GCTCGGTGTGTGCTGCTTTT |
| p38 MAPK | CCTGTAGGTCCTTTTGGCGT |
| JNK | TGTTTCCTGATGTGCTTTTCC |
| JNK | CTCCTCTATTGTGTGCTCCCT |
| ERK | ATCACCTTGTCATGGCCTCA |
| ERK | CTTGCCACTGAACTTGCCAT |

**Tab. 4. HPLC elution gradient in the chemical composition analysis of HUF extract**

| Time | Mobile phase ratio |
| --- | --- |
| 0-1 min | 8% B |
| 1-8 min | 8%-98% B |
| 8-10 min | 98% B |
| 10-10.1 min | 98%-8% B |
| 10.1-12 min | 8% B |

Mobile phase in positive ion mode: 0.1% formic acid acetonitrile (B) and 0.1% formic acid water (A). Mobile phase in negative ion mode: acetonitrile (B) and 5 mM ammonium formate water (A).

**Tab. 5. Components predicted by LC-MS in HUF**

| **No.** | **Retention time (min)** | **m/z** | **Ion mode** | **Putative Identity** | **Formula** | **Mass Error (ppm)** | **Category** |
| --- | --- | --- | --- | --- | --- | --- | --- |
| 1 | 0.695 | 973.3238 | POS | Maltohexaose | C36H62O31 | -1.0837 | Carbohydrates and Glycosides |
| 2 | 0.770 | 168.0769 | POS | Spinacine | C7H9N3O2 | 0.8084 | Amino Acids, Peptides and derivatives |
| 3 | 0.790 | 162.0762 | POS | Manosamine | C6H13NO5 | -3.1202 | Carbohydrates and Glycosides |
| 4 | 0.865 | 225.0609 | NEG | Glucose | C6H12O6 | -3.2833 | Carbohydrates and Glycosides |
| 5 | 0.897 | 387.1136 | NEG | Turanose | C12H22O11 | -2.1554 | Carbohydrates and Glycosides |
| 6 | 0.898 | 176.0107 | POS | Trigonelline | C7H7NO2 | -0.6962 | Alkaloids |
| 7 | 0.899 | 156.0420 | POS | Fosfomycin | C3H7O4P | -0.1789 | Organic acids and derivatives |
| 8 | 0.912 | 260.0528 | POS | Cyclic N-Acetyl-D-mannosamine | C8H15NO6 | -1.1861 | Carbohydrates and Glycosides |
| 9 | 0.932 | 223.0452 | NEG | 1,4-D-Gulonolactone | C6H10O6 | -3.4877 | Carbohydrates and Glycosides |
| 10 | 0.950 | 543.1319 | POS | Melezitose | C18H32O16 | -0.5491 | Carbohydrates and Glycosides |
| 11 | 0.952 | 138.0549 | POS | 3-Pyridineacetic acid | C7H7NO2 | -0.7205 | Pyridines and derivatives |
| 12 | 0.960 | 432.1705 | POS | Xylotriose | C15H26O13 | -1.6305 | Carbohydrates and Glycosides |
| 13 | 0.968 | 282.1178 | POS | Kinsenoside | C10H16O8 | -1.9613 | Carbohydrates and Glycosides |
| 14 | 0.968 | 233.0419 | POS | Methyl beta-D-Galactopyranoside | C7H14O6 | -1.3163 | Carbohydrates and Glycosides |
| 15 | 0.968 | 171.0763 | POS | Aceglutamide | C7H12N2O4 | -4.3547 | Amino Acids, Peptides and derivatives |
| 16 | 0.986 | 207.0501 | NEG | Glucoheptanoic acid | C7H14O8 | -1.7986 | Carbohydrates and Glycosides |
| 17 | 0.987 | 156.1018 | POS | Arecoline | C8H13NO2 | -0.5502 | Alkaloids |
| 18 | 1.042 | 256.0812 | POS | Nicotinic acid riboside | C11H13NO6 | -1.2676 | Carbohydrates and Glycosides |
| 19 | 1.062 | 154.0498 | POS | 5-Aminosalicylic Acid | C7H7NO3 | -0.7029 | Phenols |
| 20 | 1.098 | 217.0681 | POS | Pinitol | C7H14O6 | -0.7445 | Carbohydrates and Glycosides |
| 21 | 1.115 | 244.0925 | POS | Cytarabine | C9H13N3O5 | -1.2736 | Carbohydrates and Glycosides |
| 22 | 1.207 | 152.0566 | POS | Guanine | C5H5N5O | -0.5370 | Imidazopyrimidines |
| 23 | 1.260 | 201.1232 | POS | 4'-O-Methylpyridoxine | C9H13NO3 | -0.6057 | Pyridines and derivatives |
| 24 | 1.296 | 243.0616 | NEG | 1-beta-D-Arabinofuranosyluracil | C9H12N2O6 | -2.7365 | Carbohydrates and Glycosides |
| 25 | 1.513 | 312.0944 | NEG | Vidarabine | C10H13N5O4 | -1.6601 | Carbohydrates and Glycosides |
| 26 | 1.610 | 267.0730 | NEG | Allopurinol riboside | C10H12N4O5 | -1.7873 | Carbohydrates and Glycosides |
| 27 | 1.776 | 329.0874 | NEG | Phaseoloidin | C14H18O9 | -1.2896 | Carbohydrates and Glycosides |
| 28 | 1.776 | 219.0769 | NEG | Glycyl-L-tyrosine | C11H14N2O4 | -0.2790 | Amino Acids, Peptides and derivatives |
| 29 | 1.864 | 203.1391 | POS | Leu-ala | C9H18N2O3 | 0.3289 | Amino Acids, Peptides and derivatives |
| 30 | 2.052 | 205.0347 | NEG | Methyl citrate | C7H10O7 | -3.0968 | Carboxylic acid and derivatives |
| 31 | 2.136 | 257.0777 | NEG | 2′-O-Methyluridine | C10H14N2O6 | -1.0088 | Nucleotides and derivatives |
| 32 | 2.136 | 296.0996 | NEG | N2-Methylguanosine | C11H15N5O5 | -1.5911 | Nucleotides and derivatives |
| 33 | 2.763 | 127.0391 | POS | 5-Hydroxymethylfurfural | C6H6O3 | 0.8948 | Others |
| 34 | 2.904 | 201.1237 | NEG | Alanyleucine | C9H18N2O3 | -3.7251 | Amino Acids, Peptides and derivatives |
| 35 | 3.704 | 493.1558 | NEG | Orcinol gentiobioside | C19H28O12 | -1.0527 | Carbohydrates and Glycosides |
| 36 | 3.717 | 160.1334 | POS | 8-Aminooctanoic acid | C8H17NO2 | 0.9599 | Amino Acids, Peptides and derivatives |
| 37 | 3.801 | 124.0759 | POS | Tropone | C7H6O | 1.7521 | Others |
| 38 | 3.821 | 203.1180 | POS | Praziquanamine | C12H14N2O | 0.6421 | Organoheterocyclic compounds |
| 39 | 3.829 | 210.0766 | NEG | Polygonatine A | C9H11NO2 | -2.8273 | Others |
| 40 | 3.841 | 129.0547 | POS | Furaneol | C6H8O3 | 0.9624 | Organoheterocyclic compounds |
| 41 | 3.841 | 208.1334 | POS | Salsolidine | C12H17NO2 | 1.0141 | Organoheterocyclic compounds |
| 42 | 3.850 | 401.1107 | NEG | Sesamoside | C17H24O12 | 5.7174 | Terpenes |
| 43 | 3.872 | 331.1030 | NEG | Orcinol glucoside | C13H18O7 | -1.2544 | Carbohydrates and Glycosides |
| 44 | 3.892 | 353.0876 | NEG | Neochlorogenic acid | C16H18O9 | -0.6281 | Phenylpropanoids |
| 45 | 3.904 | 213.1232 | POS | N-acetyldopamine | C10H13NO3 | -0.8883 | Phenols |
| 46 | 3.933 | 385.0771 | NEG | Esculin | C15H16O9 | -1.2796 | Phenylpropanoids |
| 47 | 3.943 | 146.0601 | POS | 4-Aminocinnamic Acid | C9H9NO2 | -3.4868 | Phenylpropanoids |
| 48 | 3.976 | 357.1182 | NEG | Loganic acid | C16H24O10 | -0.9275 | Terpenes |
| 49 | 3.997 | 345.1190 | NEG | Salidroside | C14H20O7 | -0.3606 | Carbohydrates and Glycosides |
| 50 | 4.004 | 201.1023 | POS | Abrine | C12H14N2O2 | -2.6162 | Amino Acids, Peptides and derivatives |
| 51 | 4.044 | 311.0760 | POS | Bergenin | C14H16O9 | -2.2871 | Phenylpropanoids |
| 52 | 4.064 | 271.1150 | POS | Crenulatin | C11H20O6 | -0.6572 | Terpenes |
| 53 | 4.079 | 369.0819 | NEG | Skimmin | C15H16O8 | -2.1317 | Phenylpropanoids |
| 54 | 4.142 | 401.1083 | NEG | Cis-Ferulic acid 4-O-beta-D-glucopyranoside | C16H20O9 | -1.5397 | Carbohydrates and Glycosides |
| 55 | 4.162 | 289.1654 | NEG | Megastigm-7-ene-3,5,6,9-tetraol | C13H24O4 | -1.0647 | Terpenes |
| 56 | 4.162 | 461.1293 | NEG | Regaloside C | C18H24O11 | -1.6169 | Carbohydrates and Glycosides |
| 57 | 4.186 | 125.0599 | POS | Salicyl alcohol | C7H8O2 | 1.5970 | Phenols |
| 58 | 4.209 | 295.1287 | POS | gamma-Glu-Phe | C14H18N2O5 | -0.6729 | Amino Acids, Peptides and derivatives |
| 59 | 4.209 | 395.1311 | POS | Syringin | C17H24O9 | -0.4730 | Carbohydrates and Glycosides |
| 60 | 4.223 | 517.1559 | NEG | Sibiricose A5 | C22H30O14 | -0.7120 | Carbohydrates and Glycosides |
| 61 | 4.244 | 385.0771 | NEG | Daphnin | C15H16O9 | -1.3131 | Phenylpropanoids |
| 62 | 4.244 | 529.1556 | NEG | Sibiricose A6 | C23H32O15 | -0.2023 | Carbohydrates and Glycosides |
| 63 | 4.249 | 261.1236 | POS | Maculosin | C14H16N2O3 | 0.6978 | Amino Acids, Peptides and derivatives |
| 64 | 4.263 | 313.0926 | NEG | Glucovanillin | C14H18O8 | -0.8187 | Carbohydrates and Glycosides |
| 65 | 4.285 | 353.0875 | NEG | Scopolin | C16H18O9 | -0.7903 | Phenylpropanoids |
| 66 | 4.285 | 755.2041 | NEG | Kaempferol 3-sophoroside-7-rhamnoside | C33H40O20 | 0.0761 | Flavonoids |
| 67 | 4.290 | 291.0862 | POS | Catechin | C15H14O6 | -0.4642 | Flavonoids |
| 68 | 4.305 | 289.0715 | NEG | Epicatechin | C15H14O6 | -0.8119 | Flavonoids |
| 69 | 4.310 | 611.1606 | POS | Kaempferol 3,7-Di-o-glucoside | C27H30O16 | -0.1354 | Flavonoids |
| 70 | 4.310 | 371.0976 | POS | Fraxin | C16H18O10 | 0.8897 | Phenylpropanoids |
| 71 | 4.326 | 785.2151 | NEG | Isorhamnetin 3-sophoroside-7-rhamnoside | C34H42O21 | 0.6703 | Flavonoids |
| 72 | 4.326 | 357.1184 | NEG | Sweroside | C16H22O9 | -1.8938 | Terpenes |
| 73 | 4.370 | 154.0501 | POS | Methyl 5-hydroxypyridine-2-carboxylate | C7H7NO3 | 1.3524 | Pyridines and derivatives |
| 74 | 4.387 | 385.1136 | NEG | Oleoside 11-methyl ester | C17H24O11 | 0.2141 | Terpenes |
| 75 | 4.387 | 367.1026 | NEG | 3-O-Caffeoylquinic acid methyl ester | C17H20O9 | -2.2438 | Phenylpropanoids |
| 76 | 4.390 | 237.0735 | POS | 3,4-O-Isopropylidene-shikimic acid | C10H14O5 | 0.5785 | Others |
| 77 | 4.408 | 461.1109 | NEG | Puerarin | C21H20O9 | 4.3498 | Flavonoids |
| 78 | 4.428 | 459.1136 | NEG | Asperuloside | C18H22O11 | -1.6970 | Terpenes |
| 79 | 4.428 | 243.1710 | NEG | Leu-Leu | C12H24N2O3 | -1.6615 | Amino Acids, Peptides and derivatives |
| 80 | 4.450 | 387.1291 | NEG | Verbenalin | C17H24O10 | -1.5276 | Terpenes |
| 81 | 4.450 | 207.0293 | NEG | 7-Hydroxychromone | C9H6O3 | -2.9847 | Phenols |
| 82 | 4.454 | 241.0950 | POS | 1-Methyl-L-tryptophan | C12H14N2O2 | 0.9271 | Amino Acids, Peptides and derivatives |
| 83 | 4.474 | 469.1681 | POS | Cimifugin 4'-O-beta-D-glucopyranoside | C22H28O11 | -4.8865 | Carbohydrates and Glycosides |
| 84 | 4.491 | 771.1993 | NEG | Camelliaside B | C32H38O19 | 0.4624 | Flavonoids |
| 85 | 4.491 | 737.1937 | NEG | Camelliaside A | C33H40O20 | 1.0579 | Flavonoids |
| 86 | 4.515 | 327.1424 | POS | Raspberry ketone glucoside | C16H22O7 | -4.2477 | Carbohydrates and Glycosides |
| 87 | 4.534 | 431.1916 | NEG | Roseoside | C19H30O8 | -1.4726 | Terpenes |
| 88 | 4.534 | 237.0401 | NEG | 4-Methylesculetin | C10H8O4 | -1.3801 | Phenylpropanoids |
| 89 | 4.554 | 177.0186 | NEG | Daphnetin | C9H6O4 | -4.3656 | Phenylpropanoids |
| 90 | 4.574 | 605.1853 | NEG | Magnoloside A | C29H36O15 | -2.8976 | Carbohydrates and Glycosides |
| 91 | 4.574 | 727.2455 | NEG | Pinoresinol Diglucoside | C32H42O16 | -0.0279 | Phenylpropanoids |
| 92 | 4.574 | 591.1352 | NEG | Panasenoside | C27H30O16 | 0.3469 | Flavonoids |
| 93 | 4.578 | 757.2186 | POS | Kaempferol-3-o-(2"-o-beta-d- glucopyl)-beta-d–rutinoside | C33H40O20 | 0.0528 | Flavonoids |
| 94 | 4.598 | 469.1318 | POS | Gaultherin | C19H26O12 | 0.3284 | Carbohydrates and Glycosides |
| 95 | 4.598 | 399.1413 | POS | Ailanthone | C20H24O7 | -0.4114 | Terpenes |
| 96 | 4.636 | 505.1561 | NEG | Apiopaeonoside | C20H28O12 | -0.2631 | Carbohydrates and Glycosides |
| 97 | 4.705 | 741.2241 | POS | Grosvenorine | C33H40O19 | 0.5561 | Flavonoids |
| 98 | 4.705 | 153.0549 | POS | Isovanillin | C8H8O3 | 1.6945 | Phenols |
| 99 | 4.726 | 369.1182 | POS | Methyl chlorogenate | C17H20O9 | 0.6175 | Phenylpropanoids |
| 100 | 4.746 | 771.2346 | POS | Typhaneoside | C34H42O20 | 0.4355 | Flavonoids |
| 101 | 4.761 | 523.2186 | NEG | Secoisolariciresinol monoglucoside | C26H36O11 | 0.2265 | Phenylpropanoids |
| 102 | 4.761 | 429.1399 | NEG | Barlerin | C19H28O12 | 0.5650 | Terpenes |
| 103 | 4.804 | 519.1139 | NEG | Sibiricaxanthone B | C24H26O14 | 0.0317 | Phenylpropanoids |
| 104 | 4.804 | 547.1106 | NEG | Daidzin 6′′-O-malonate | C24H22O12 | 2.3699 | Flavonoids |
| 105 | 4.830 | 208.0971 | POS | 6-Benzylaminopurine | C12H11N5 | -8.1191 | Imidazopyrimidines |
| 106 | 4.845 | 609.1457 | NEG | Quercetin-3-O-D-glucosyl]-(1-2)-L-rhamnoside | C27H30O16 | -0.7182 | Flavonoids |
| 107 | 4.845 | 639.1566 | NEG | Biorobin | C27H30O15 | -0.1582 | Flavonoids |
| 108 | 4.845 | 433.1133 | NEG | Isohemiphloin | C21H22O10 | -1.7594 | Flavonoids |
| 109 | 4.845 | 224.0923 | NEG | Fusaric acid | C10H13NO2 | -2.3627 | Pyridines and derivatives |
| 110 | 4.850 | 307.1151 | POS | Phenylethyl-beta-D-galactoside | C14H20O6 | -0.4880 | Carbohydrates and Glycosides |
| 111 | 4.850 | 154.0864 | POS | m-Anisaldehyde | C8H8O2 | 0.8967 | Others |
| 112 | 4.888 | 565.1924 | NEG | Pinoresinol 4-O-beta-D-glucopyranoside | C26H32O11 | -0.4039 | Phenylpropanoids |
| 113 | 4.888 | 755.2040 | NEG | Complanatoside B | C33H40O20 | -0.0550 | Flavonoids |
| 114 | 4.934 | 223.0600 | POS | Isofraxidin | C11H10O5 | -0.2312 | Phenylpropanoids |
| 115 | 4.956 | 303.0496 | POS | Robinetin | C15H10O7 | -1.0498 | Flavonoids |
| 116 | 4.970 | 579.1353 | NEG | Graveobioside A | C26H28O15 | -0.3970 | Flavonoids |
| 117 | 4.970 | 461.0720 | NEG | Cannabiscitrin | C21H20O13 | 0.0012 | Flavonoids |
| 118 | 4.976 | 403.1371 | POS | Rhapontigenin 3'-o-glucoside | C21H24O9 | -5.5364 | Phenylpropanoids |
| 119 | 4.990 | 593.1507 | NEG | Kaempferol-3-o-beta-d-glucosyl(1-2)rhamnoside | C27H30O15 | -0.7515 | Flavonoids |
| 120 | 4.990 | 899.2253 | NEG | Astilbin | C21H22O11 | 0.1628 | Flavonoids |
| 121 | 4.996 | 595.1659 | POS | Nicotiflorin | C27H30O15 | 0.3153 | Flavonoids |
| 122 | 4.996 | 163.0752 | POS | Coniferyl alcohol | C10H12O3 | -4.3615 | Phenols |
| 123 | 5.017 | 433.1129 | POS | Genistin | C21H20O10 | -0.0893 | Flavonoids |
| 124 | 5.017 | 207.1379 | POS | Vomifoliol | C13H20O3 | -3.0738 | Terpenes |
| 125 | 5.017 | 216.1229 | POS | Guaifenesin | C10H14O4 | -0.6031 | Others |
| 126 | 5.036 | 463.1230 | POS | Tectoridin | C22H22O11 | -1.1406 | Flavonoids |
| 127 | 5.052 | 623.1611 | NEG | Narcissin | C28H32O16 | -0.9949 | Flavonoids |
| 128 | 5.052 | 419.1340 | NEG | Rhapontin | C21H24O9 | -1.7183 | Phenylpropanoids |
| 129 | 5.056 | 469.0786 | POS | Paederoside | C18H22O11S | 2.3282 | Terpenes |
| 130 | 5.115 | 603.2048 | POS | Acanthoside B | C28H36O13 | -0.0258 | Phenylpropanoids |
| 131 | 5.115 | 153.0545 | POS | 2-Methoxybenzoic acid | C8H8O3 | -0.5469 | Others |
| 132 | 5.115 | 130.0651 | POS | Indole-3-carbinol | C9H9NO | -4.5158 | Indoles and derivatives |
| 133 | 5.135 | 601.1549 | NEG | Camellianin A | C29H32O15 | -1.4412 | Flavonoids |
| 134 | 5.135 | 247.0986 | NEG | Guaiacol | C7H8O2 | 4.0955 | Phenylpropanoids |
| 135 | 5.153 | 449.1075 | POS | Trifolin | C21H20O11 | -0.7525 | Flavonoids |
| 136 | 5.153 | 287.0546 | POS | Dihydromorin | C15H12O7 | -3.3609 | Flavonoids |
| 137 | 5.173 | 177.0546 | POS | Isoferulic acid | C10H10O4 | -3.6486 | Phenylpropanoids |
| 138 | 5.173 | 312.1224 | POS | N-Feruloyloctopamine | C18H19NO5 | -4.0657 | Phenylpropanoids |
| 139 | 5.173 | 207.0652 | POS | Sinapinic acid | C11H12O5 | -2.8076 | Phenylpropanoids |
| 140 | 5.192 | 183.0651 | POS | Veratric acid | C9H10O4 | -0.4495 | Others |
| 141 | 5.210 | 479.1181 | POS | Brassicin | C22H22O12 | -0.5671 | Flavonoids |
| 142 | 5.210 | 317.0651 | POS | Azaleatin | C16H12O7 | -1.6440 | Flavonoids |
| 143 | 5.216 | 303.0504 | NEG | Taxifolin | C15H12O7 | -2.0134 | Flavonoids |
| 144 | 5.216 | 243.0295 | NEG | Urolithin C | C13H8O5 | -1.7381 | Phenylpropanoids |
| 145 | 5.256 | 461.0723 | NEG | Luteolin-3-O-beta-D-glucuronide | C21H18O12 | -0.5634 | Flavonoids |
| 146 | 5.268 | 167.0703 | POS | Apocynin | C9H10O3 | 0.3574 | Phenols |
| 147 | 5.268 | 271.0599 | POS | 7,3',4'-trihydroxyflavone | C15H10O5 | -0.6456 | Flavonoids |
| 148 | 5.276 | 577.1555 | NEG | Rubrofusarin gentiobioside | C27H32O15 | -0.4009 | Phenylpropanoids |
| 149 | 5.310 | 419.0970 | POS | Kaempferol 3-alpha-L-arabinopyranoside | C20H18O10 | -0.7584 | Flavonoids |
| 150 | 5.359 | 559.1455 | NEG | Chrysin 7-O-beta-gentiobioside | C27H30O14 | 0.5394 | Flavonoids |
| 151 | 5.381 | 503.1194 | NEG | Iridin | C24H26O13 | 0.9257 | Flavonoids |
| 152 | 5.381 | 563.1045 | NEG | Apigenin-7-O-6''-Malonylglucoside | C24H22O13 | 0.4472 | Flavonoids |
| 153 | 5.394 | 209.0810 | POS | Sinapaldehyde | C11H12O4 | 0.7405 | Phenylpropanoids |
| 154 | 5.402 | 298.1081 | NEG | N-trans-caffeoyltyramine | C17H17NO4 | -1.2694 | Phenylpropanoids |
| 155 | 5.414 | 357.1324 | POS | Coniferyl ferulic acid | C20H20O6 | -2.3534 | Phenylpropanoids |
| 156 | 5.464 | 429.0820 | NEG | Vincetoxicoside B | C21H20O11 | -0.2881 | Flavonoids |
| 157 | 5.464 | 307.0823 | NEG | Rutaretin | C14H14O5 | -0.0215 | Phenylpropanoids |
| 158 | 5.476 | 149.0599 | POS | Methyl 4-hydroxyphenylacetate | C9H10O3 | -2.7228 | Phenols |
| 159 | 5.485 | 491.1195 | NEG | Aurantio-obtusin beta-D-glucoside | C23H24O12 | -0.0735 | Phenylpropanoids |
| 160 | 5.497 | 233.0760 | POS | Kifunensine | C8H12N2O6 | -3.4420 | Amino Acids, Peptides and derivatives |
| 161 | 5.497 | 429.1169 | POS | 4''-methyloxy-Genistin | C22H22O10 | -4.0691 | Flavonoids |
| 162 | 5.517 | 152.0708 | POS | Phthalide | C8H6O2 | 1.2644 | Steroids |
| 163 | 5.549 | 433.1139 | NEG | Isoengelitin | C21H22O10 | -0.3577 | Flavonoids |
| 164 | 5.569 | 521.1662 | NEG | Oleuroside | C25H32O13 | 0.6322 | Terpenes |
| 165 | 5.569 | 319.0852 | NEG | Coptisine | C19H14NO4+ | 2.1875 | Alkaloids |
| 166 | 5.582 | 207.0653 | POS | 7-Methoxy-4-methyl-coumarin-8-ol | C11H10O4 | 0.6599 | Phenylpropanoids |
| 167 | 5.582 | 385.1619 | POS | Secoisolariciresinol | C20H26O6 | -0.5550 | Phenylpropanoids |
| 168 | 5.611 | 417.1184 | NEG | Neoisoliquiritin | C21H22O9 | -1.6002 | Carbohydrates and Glycosides |
| 169 | 5.611 | 351.0717 | NEG | Gallocatechin | C15H14O7 | -1.2165 | Flavonoids |
| 170 | 5.631 | 431.0978 | NEG | Oroxin A | C21H20O10 | -1.4021 | Flavonoids |
| 171 | 5.631 | 475.1597 | NEG | 6'-O-cinnamoyl harpagide | C24H30O11 | -1.5134 | Carbohydrates and Glycosides |
| 172 | 5.667 | 531.1493 | POS | 4,5-Di-O-caffeoylquinic acid methyl ester | C26H26O12 | -0.7524 | Phenylpropanoids |
| 173 | 5.686 | 179.0703 | POS | Coniferaldehyde | C10H10O3 | -0.0884 | Phenylpropanoids |
| 174 | 5.691 | 287.0556 | NEG | Okanin | C15H12O6 | -1.6068 | Flavonoids |
| 175 | 5.708 | 246.1124 | POS | Resveratrol | C14H12O3 | -0.1712 | Phenylpropanoids |
| 176 | 5.769 | 227.0814 | POS | Methyl beta-carboline-1-carboxylate | C13H10N2O2 | -0.2948 | Alkaloids |
| 177 | 5.794 | 193.0498 | NEG | Propyl gallate | C10H12O5 | -1.5713 | Phenols |
| 178 | 5.856 | 403.1395 | NEG | Desoxyrhaponticin | C21H24O8 | -0.7511 | Phenylpropanoids |
| 179 | 5.897 | 167.0704 | POS | Veratraldehyde | C9H10O3 | 0.4942 | Others |
| 180 | 5.918 | 483.2361 | NEG | Segetalin B | C24H32N6O5 | -0.0229 | Flavonoids |
| 181 | 5.918 | 227.1283 | NEG | 2-Methyl-2-pentenoic acid | C6H10O2 | -2.4689 | Fatty Acyls |
| 182 | 6.003 | 327.2148 | POS | Aleuritic acid | C16H32O5 | 1.9315 | Fatty Acyls |
| 183 | 6.003 | 209.0809 | POS | 3,4-Dimethoxycinnamic acid | C11H12O4 | 0.4526 | Phenylpropanoids |
| 184 | 6.021 | 373.1292 | NEG | (+)-Nortrachelogenin | C20H22O7 | -0.2046 | Phenylpropanoids |
| 185 | 6.063 | 475.0875 | NEG | Chrysin-7-O-glucuronide | C21H18O10 | -1.4329 | Flavonoids |
| 186 | 6.104 | 363.1810 | NEG | Oridonin | C20H28O6 | -0.9059 | Terpenes |
| 187 | 6.146 | 505.0984 | NEG | Oroxylin A-7-O-glucuronide | C22H20O11 | -0.6387 | Flavonoids |
| 188 | 6.146 | 329.0661 | NEG | Glycitein | C16H12O5 | -1.5996 | Flavonoids |
| 189 | 6.187 | 321.0869 | NEG | 3-Aminocoumarin | C9H7NO2 | -3.7463 | Phenylpropanoids |
| 190 | 6.187 | 231.1597 | NEG | 3-Methylvaleric Acid | C6H12O2 | -2.0651 | Fatty Acyls |
| 191 | 6.187 | 287.0559 | NEG | Steppogenin | C15H12O6 | -0.7780 | Flavonoids |
| 192 | 6.291 | 301.0349 | NEG | Morin | C15H10O7 | -1.5533 | Flavonoids |
| 193 | 6.297 | 303.0496 | POS | Quercetin | C15H10O7 | -1.0189 | Flavonoids |
| 194 | 6.338 | 401.1591 | POS | Syringaresinol | C22H26O8 | -2.4900 | Phenylpropanoids |
| 195 | 6.466 | 309.0866 | POS | Flazin | C17H12N2O4 | -1.3032 | Alkaloids |
| 196 | 6.466 | 371.1484 | POS | Medioresil | C21H24O7 | -3.0488 | Phenylpropanoids |
| 197 | 6.499 | 209.0809 | POS | Ethyl Caffeic acid | C11H12O4 | 0.2417 | Phenylpropanoids |
| 198 | 6.559 | 223.0971 | NEG | Methyl isoeugenol | C11H14O2 | -2.2929 | Phenylpropanoids |
| 199 | 6.559 | 445.1136 | NEG | Prunetrin | C22H22O10 | -0.8891 | Flavonoids |
| 200 | 6.591 | 357.1326 | POS | (+)-Balanophonin | C20H20O6 | -1.9387 | Phenylpropanoids |
| 201 | 6.633 | 196.0968 | POS | 4-Methoxycinnamic acid | C10H10O3 | 0.0587 | Phenylpropanoids |
| 202 | 6.633 | 168.1019 | POS | 4-Allylcatechol | C9H10O2 | 0.0248 | Phenylpropanoids |
| 203 | 6.742 | 245.0808 | POS | p-hydroxy-5,6-dehydrokawain | C14H12O4 | -0.1393 | Phenols |
| 204 | 6.925 | 383.1509 | POS | Neoanhydropodophyllol | C22H24O7 | 3.5348 | Phenylpropanoids |
| 205 | 6.966 | 271.0609 | NEG | Pinobanksin | C15H12O5 | -1.0156 | Flavonoids |
| 206 | 7.046 | 849.3741 | NEG | Rebaudioside G | C38H60O18 | -2.4780 | Terpenes |
| 207 | 7.136 | 285.0399 | NEG | Kaempferol | C15H10O6 | -1.8162 | Flavonoids |
| 208 | 7.313 | 315.0506 | NEG | Tamarixetin | C16H12O7 | -1.3695 | Flavonoids |
| 209 | 7.477 | 269.0454 | NEG | Baicalein | C15H10O5 | -0.4601 | Flavonoids |
| 210 | 7.530 | 164.1069 | POS | Benzylideneacetone | C10H10O | -0.2462 | Phenylpropanoids |
| 211 | 7.551 | 182.1176 | POS | 2-Phenylbutanoic acid | C10H12O2 | 0.0266 | Others |
| 212 | 7.581 | 299.0558 | NEG | 3,4'-Dihydroxyflavone | C15H10O4 | -0.9526 | Flavonoids |
| 213 | 7.622 | 249.1482 | POS | Ivangustin | C15H20O3 | -1.1045 | Terpenes |
| 214 | 7.700 | 298.1433 | POS | Aegeline | C18H19NO3 | -1.5200 | Phenylpropanoids |
| 215 | 7.909 | 821.3965 | NEG | Glycyrrhizic acid | C42H62O16 | -0.0078 | Terpenes |
| 216 | 7.951 | 267.0658 | NEG | Poriol | C16H14O5 | 0.1526 | Flavonoids |
| 217 | 7.963 | 269.0805 | POS | Dalbergin | C16H12O4 | -1.1820 | Phenylpropanoids |
| 218 | 8.058 | 195.1379 | POS | 3-oxo-2-pentylcyclopentaneacetic acid | C12H20O3 | -3.1766 | Fatty Acyls |
| 219 | 8.100 | 659.1417 | NEG | Aurantio-obtusin | C17H14O7 | 1.6284 | Phenylpropanoids |
| 220 | 8.113 | 155.1066 | POS | Ethyl pivaloylacetate | C9H16O3 | -4.2312 | Organic acids and derivatives |
| 221 | 8.256 | 315.0495 | NEG | isorhamnetin | C16H12O7 | -1.9278 | Flavonoids |
| 222 | 8.482 | 531.1797 | NEG | 6-Hydroxy-2-(2-phenylethyl)chromone | C17H14O3 | -3.0352 | Flavonoids |
| 223 | 8.498 | 779.4212 | POS | Anemarrhenasaponin III | C39H64O14 | 3.0049 | Steroids |
| 224 | 8.656 | 413.1592 | NEG | Pseudolaric Acid B | C23H28O8 | -1.9225 | Terpenes |
| 225 | 8.656 | 243.1597 | NEG | Tridecanedioic acid | C13H24O4 | -1.8360 | Fatty Acyls |
| 226 | 8.978 | 277.1795 | POS | [6]-Gingerol | C17H26O4 | -3.3221 | Phenols |
| 227 | 9.090 | 427.1758 | NEG | Yangambin | C24H30O8 | 0.2490 | Phenylpropanoids |
| 228 | 9.533 | 299.2001 | POS | 15-Hydroxydehydroabietic acid | C20H28O3 | -3.3919 | Terpenes |
| 229 | 9.554 | 225.1484 | POS | Methyljasmonate | C13H20O3 | -0.5717 | Fatty Acyls |
| 230 | 9.881 | 601.3016 | NEG | Cucurbitacin E | C32H44O8 | -0.3770 | Terpenes |
| 231 | 9.965 | 215.1648 | NEG | 3-Hydroxydodecanoic acid | C12H24O3 | -2.3575 | Organic acids and derivatives |
| 232 | 10.841 | 327.0776 | POS | Triphenyl phosphate | C18H15O4P | -1.2936 | Organic acids and derivatives |
| 233 | 11.002 | 233.1542 | NEG | Artemisic acid | C15H22O2 | -2.1899 | Terpenes |
| 234 | 11.720 | 343.0816 | NEG | Usnic acid | C18H16O7 | -2.0427 | Phenols |
| 235 | 12.797 | 256.2633 | POS | Bombykol | C16H30O | -0.7055 | Fatty Acyls |

*Identifications are putative and based on MS/MS spectral library matching (Level 2 confidence); authentic standards were not used for confirmation unless otherwise noted.
